# Telenursing Health Education and Lifestyle Modification Among Patients With Diabetes in Bangladesh: Protocol for a Pilot Study With a Quasi-experimental Pre- and Postintervention Design

**DOI:** 10.2196/71849

**Published:** 2025-05-09

**Authors:** Michiko Moriyama, K A T M Ehsanul Huq, Lucy Mondol, Akhi Roy Mita, Niru Shamsun Nahar

**Affiliations:** 1 Graduate School of Biomedical and Health Sciences Hiroshima University Hiroshima Japan; 2 Department of Nursing Grameen Caledonian College of Nursing Dhaka Bangladesh

**Keywords:** telenursing, health education, lifestyle modification, diabetic patients, Bangladesh

## Abstract

**Background:**

The global burden of chronic diseases is increasing and becoming a public health issue throughout the world. The use of telenursing is increasing significantly during and after the COVID-19 pandemic to treat and prevent chronic diseases. Telenursing is growing in many countries to reduce health care costs, increase the number of aging and chronically ill populations, and increase health care coverage to distant, rural, small, or sporadically populated regions. Among its many benefits, telenursing may help to solve increasing shortages of nurses, reduce distances, save travel time, and keep patients out of the hospital.

**Objective:**

The objective of this study is to apply the self-management telenursing program and telenursing system developed by the researchers to Bangladesh and to evaluate its feasibility and efficacy (improved diabetes control in participants).

**Methods:**

This is a pilot, quasi-experimental pre- and post-intervention study. Diabetes patients who will attend the Grameen Primary Health Centers (PHCs) in Bangladesh will be enrolled between September 2024 and August 2025. We include patients who have been diagnosed with type 2 diabetes, both sexes, ages 18-75 years old, all types of treatment, willing to participate and give us consent. We exclude patients who have been diagnosed with gestational diabetes, diabetes as a secondary cause, complication of chronic kidney disease (CKD) stage 5, Hemoglobin A1c (HbA_1c_) is less than 7% for the past 1 year with CKD stage 1 or 2, no complications or complications with good control, having enough knowledge (had education before) and implemented good practice regarding diabetes management assessed by the research nurses, and disabled persons who need other person’s support for daily living. The sample size was calculated and found 70. Written informed consent will be obtained from all the participants. The study protocol got approval from the National Research Ethics Committee of the Bangladesh Medical Research Council (BMRC/NREC/2022-2025/336) on September 08, 2024. The outcome of this study is to evaluate the effects of telenursing intervention by controlling HbA_1c_.

**Results:**

The project was funded in 2024. The enrollment of the participants started on October 26, 2024, and the required sample (n=70) enrollment was completed in February 2025. Data analysis will be started after completion of data collection and results will be expected to be submitted for publication in 2026.

**Conclusions:**

Diabetic patients will acquire disease-specific management skills. Setting and monitoring goals ensures the continuation of the desired behavior and gives the patients control over their lifestyle. After developing self-management skills, patients assess their lab data and lifestyles including diet, and understand their condition so that they can work with their physiological data by acquiring knowledge of both the disease and self-care. By making self-supported decisions, the patients will be able to manage their diet, exercise, and medication.

**Trial Registration:**

ClinicalTrials.gov NCT06632652; https://clinicaltrials.gov/study/NCT06632652

**International Registered Report Identifier (IRRID):**

DERR1-10.2196/71849

## Introduction

### Background

The global burden of noncommunicable diseases (NCDs) is increasing and becoming a public health issue throughout the world [1]. Globally, diabetes caused 1.6 million deaths and 47% of all deaths before the age of 70 years in 2021. Diabetes increases the risk of death from kidney and cardiovascular diseases [2]. The prevalence of diabetes has been increasing over the last few decades in all countries and is considered a global epidemic [3], more quickly in low- and middle-income (LMICs) countries than in high-income countries. Moreover, diabetic treatment coverage was the lowest in the low- and middle-income range in 2022 [4]. It is alarming that an estimated half of the global people (49.7%) living with diabetes remain undiagnosed [5]. Diabetes has acute complications including ketoacidosis, hypoglycemia, or hyperglycemia. Chronic complications are macroangiopathy, retinopathy, nephropathy, neuropathy, and diabetic foot. Patients with diabetes are vulnerable to infections, myopathy, osteoporosis, arthropathies, and liver damage [6]. Patients with diabetes may be able to prevent or delay diabetic health problems by maintaining a healthy lifestyle, taking medicine regularly, and controlling blood glucose levels [7].

The use of telenursing is increasing significantly during and after the COVID-19 pandemic to treat and prevent chronic diseases [8]. As a field, it is part of telemedicine and has many points of contact with other medical and nonmedical applications, such as telediagnosis, teleconsultation, and telemonitoring. The field, however, is still being developed as the information on telenursing is not comprehensive enough [9].

Telenursing has been adopted by many developed and developing countries. It reduces health care costs and increases the coverage of health care to outreach, rural, small, or sporadically populated regions [10]. It is useful for the increasing number of aging and chronically ill populations [11]. Among its many benefits, telenursing may help solve increasing shortages of nurses [12]; to reduce distances and save travel time and keep patients out of hospital [10]. A greater degree of job satisfaction has been registered among telenurses [13]. Continuous glucose monitoring (CGM) in patients with diabetes by integrating diagnostic devices for 24-hour real-time data transmission [14] and IBM Watson Health for personalized lifestyle optimization can help to identify and prevent the early appearance of diseases. It allows rural patients to receive personalized, high-quality care and improves health care access and outcomes [15].

### Diabetes in Bangladesh

The prevalence of diabetes was 9.2% (male: 8.8%, female: 9.6%) and prediabetes was 13.3% (male: 13%, female: 13.6%) in 2017. Among the patients, only 30.4% had controlled diabetes, 35.2% took treatment regularly and 61.5% were not aware of their diabetic condition. The factors related to getting diabetes were increasing age, overweight, and hypertension. The proportional mortality was 3% from diabetes of total death in all ages. In Bangladesh, 17% population was overweight (male: 14.4%, female: 19.6%), 3.3% were obese (male: 2%, female: 4.6%), and 25.1% had physical inactivity (male: 9.2%, female: 41.3%), those are the related risk factors for diabetes [16]. For the national response to diabetes, Bangladesh has an operational policy, strategy, and action plan for controlling diabetes and reducing physical inactivity, but not for reducing overweight and obesity. There are evidence-based national diabetes guidelines available, but not fully implemented. There are no set standard criteria for referral of patients from primary care to higher levels of care. In the primary care facilities, there are only blood glucose measurements and urine strips for glucose and ketone measurements generally available, but no facilities for insulin, metformin, sulphonylurea, oral glucose tolerance test, hemoglobin A_1c_ (HbA_1c_) test, dilated fundus examination, retinal photocoagulation, foot vibration perception by tuning fork, renal replacement therapy by dialysis, foot vascular status by Doppler, renal replacement therapy by transplantation in 2016 [17].

As there are no standard criteria for referring patients from primary care to a higher level of care, we will facilitate the patients to acquire self-management skills to understand the risk factors for diabetic complications and seek treatment in referral hospitals as necessary. They can attend referral hospitals to prevent diabetic complications by doing HbA_1c_ tests, dilated fundus examinations, retinal photocoagulation, foot vibration perception by tuning fork, renal replacement therapy by dialysis and transplantation, and also foot vascular status by Doppler.

### Study Objectives

#### General Objective

To apply the self-management telenursing program and telenursing system developed by the researchers to Bangladesh to evaluate its feasibility and efficacy (improved diabetes control in participants)

#### Specific Objectives

- To control patients’ diabetic condition by measuring HbA_1c_.
- By having them acquire self-management behaviors and increase their self-efficacy, to minimize the risk of arising diabetic complications.
- To see the feasibility and barriers to implementing telenursing among patients with diabetes in Bangladesh.

#### Research Questions

Whether implementing a health education program will improve diabetic patient outcomes or not and will be operational and acceptable by the patients and health care professionals in Bangladesh.

#### Hypothesis

1. The diabetes self-management program developed by the researchers cannot improve the participants’ management of their diabetes (avoid aggravation, develop complications, and hospitalization) (H_0_) or can improve the participants’ management of their diabetes (H_1_).
2. Is the telenursing system developed by the researchers operational in Bangladesh? Can the system be used by health care personnel (nurses) and patients? What are the obstacles and challenges, if any, in using the system?
3. Is the diabetes self-management program developed by researchers acceptable to health care providers and patients? How about engagement? Will the program be continued (will there be dropouts)? If so, what are the reasons?

## Methods

### Design and Period

A pilot study with a quasi-experimental pre- and postintervention design will be implemented. As we will try to explore the participants’ characteristics, we want to conduct a pilot study to see the feasibility of this project among these participants. We plan to conduct quasi-experimental pre- and postintervention design, as we found in this setting randomization; case-control is rather difficult. Therefore, we will enroll the participants and collect baseline data and then we will educate them, and after 6 months we will evaluate them and compare the baseline (pretest) and the endline (posttest) data of individual participants. We set 6 months, as a previous study showed that a patient’s behavioral modification requires at least 6 months of intervention [18]. The study duration will be 1 year after getting approval from the Ethical Committee.

### Study Participants

The participants are people with diabetes, who take services from Grameen primary health centers (PHCs). We use 2 PHCs from Sahorail and Jamshahat of Manikganj District in Bangladesh.

#### Inclusion Criteria

The inclusion criteria are as follows:

- Having been diagnosed with type 2 diabetes
- Being of either sex
- Being 18-75 years
- Having a smartphone or mobile phone
- Willingness to participate and give us consent

#### Exclusion Criteria

The exclusion criteria are as follows:

- Patients who have been diagnosed as type 1 and gestational diabetes
- Patients whose diabetes are secondary causes
- Patients who have complications of chronic kidney disease (CKD) stage 5
- Patients whose HbA_1c_ has been less than 7% for the past 1 year with CKD stage 1 or 2, with no complications or complications with good control, have enough knowledge (had education before) and implemented good practice regarding diabetes management assessed by the research nurses (hereafter “the nurses”)
- Disabled persons who need other people’s support for daily living

### Sampling Technique

We will use a purposive sampling technique to select the participants. Patients who meet the eligibility criteria will be introduced by physicians at the PHCs, and the nurses will contact them at the PHC and obtain their consent.

### Sample Size

We calculated the sample size using G*Power software (version 3.1.9.7; Heinrich-Heine-University). As this is a pilot study, we set an effect size of 0.39 considering the differences between 2 dependent matched pair means of HbA_1c_ from a previous study [19], with a level of significance of 5%, and 80% power, the total sample size is calculated at 54. As patients have many options to discontinue the study (consent, withdraw, migration, change the health care facilities based on the disease severity, complications, hospitalization, etc), we consider a 30% dropout (16 participants) for this study. Therefore, the total sample size is estimated to be 70 (Textbox 1).

**Output of software after generating sample size calculation.**

Effect size dz = 0.39

α err prob = 0.05

Input: Tail(s) = 2

Power (1-β err prob) = 0.80

Output: Noncentrality parameter δ = 2.8659030

Critical *t*= 2.0057460

Df = 53

Total sample size = 54

Actual power = 0.8033525

### Method of Data Collection Procedures

The principal investigator (PI) and co-investigators (telenurses) received training in telenursing such as communication and behavior modification techniques, and self-management skills on diabetes including blood sugar control and foot care. They will receive study-specific training from other coinvestigators and one diabetic consultant on data collection such as how to collect data and what type of tool has to be used during data collection. The patients who will attend the PHCs and telenurses will identify the patients with diabetes by checking the patient’s blood test report. They will check the participants’ eligibility criteria (inclusion and exclusion criteria) and obtain written informed consent. It will be ensured that there will be no risk in disclosing the information.

They will educate patients using an investigator-developed “Education Booklet” at the PHCs. The booklet will be given to the patients after receiving consent and health education will be provided at the time as scheduled (Multimedia Appendices 1 and 2). The data will be collected within a 6-month study period using:

1. “Initial assessment sheet” developed by the investigators. It will be filled out once by the telenurses in the PHCs to assess the patient’s initial condition and to set the goals for their behavior change. This contains sociodemographic, clinical history, and lifestyle.
2. “Self-monitoring book” developed by the investigators (Multimedia Appendix 3). Patients will complete their daily life activities including their laboratory investigation reports at home. The telenurses will educate and guide them on how to fill it over telecommunication. This contains daily practice goals (eg, energy intake, salt, exercise, ideal weight), laboratory data record (eg HbA_1c_, fasting blood sugar [FBS] or random blood sugar [RBS], lipid profile, and renal function), foot care (monthly check), daily recording (RBS if needed, blood pressure, medication intake, daily goal achievement of diet and exercise) and behavior change goals (monthly).
3. “Diabetes management self-efficacy scale” developed by Bijl et al [20] and has 20 items with a 5-point Likert scale from 1-5. The scores range from 20 to 100, a higher point indicates better self-efficacy.
4. The “Lifestyle behavior questionnaire” developed by the investigators has 6 items with a 6-point Likert scale from 0 to 5, a higher point indicates a better lifestyle. This is asked every month to check their behavior by the telenurse.
5. “Patient Satisfaction” on tele-education and prepared materials developed by the investigators. It has 6-item with a 6-point Likert Scale, and higher indicates more satisfaction.
6. A “Monthly outcome checklist” was developed by the investigators (Multimedia Appendix 4). The telenurses interview patients monthly asking about education material use, goal attainment, and complication management. The engagement rate and follow-up rate also will be counted every month by the telenurses.

All of the above except Diabetes management self-efficacy scale (number 3), was reviewed by the investigator team with the telenursing, diabetes, and chronic care nursing specialists for validity.

Data on all complications of diabetes will be asked of the patients by the investigators and check the accuracy of the information from the records.

### Research Collaboration Framework

**Figure 1:**
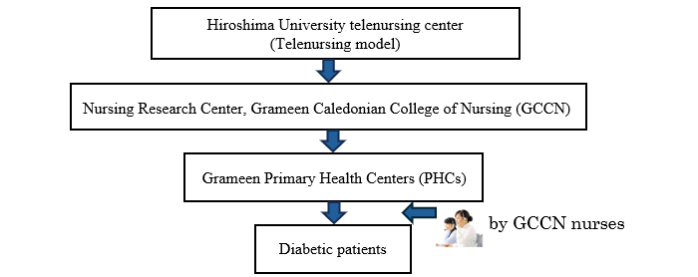
Research collaboration framework.

We will use the telenursing model with the collaboration of the Hiroshima University telenursing center and the Nursing Research Center of Grameen Caledonian College of Nursing (GCCN) (Figure 1).

### Intervention

#### Operational Definition of Self-Management and Structure of the Self-Management Program

**Figure 2:**
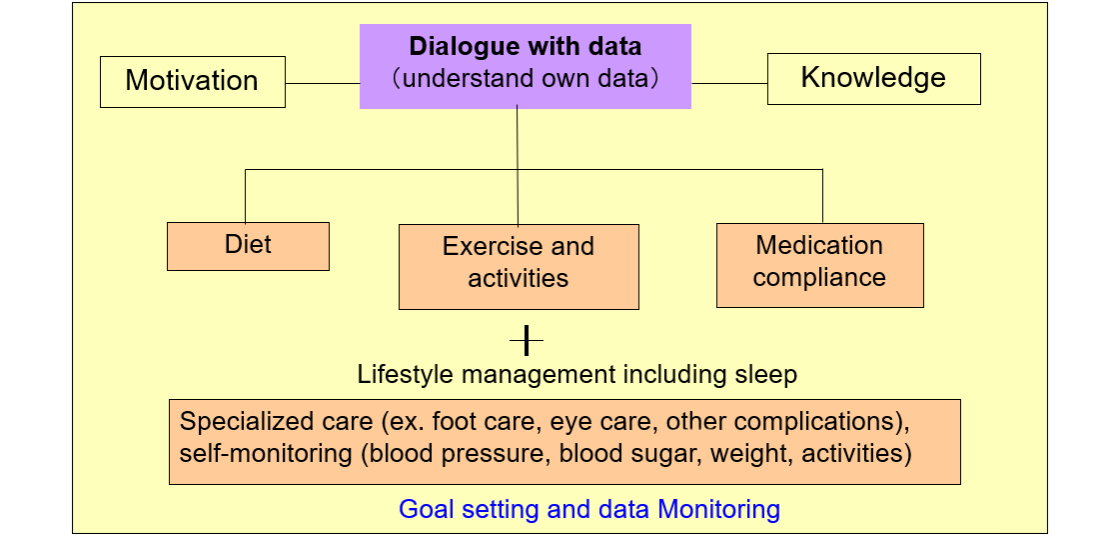
Structure of the self-management program.

The focus of this program is the acquisition of self-management skills, operationally defined in Figure 2. First, the patients analyze their laboratory data and lifestyles including diet, and understand their condition so that they can work with their physiological data by acquiring knowledge of both the disease and self-care. By making self-supported decisions, the patients can manage their diet, exercise, and medication. They also acquire disease-specific management skills. Setting goals and then monitoring them ensures the continuation of the desired behavior and gives the patients control over their lifestyle [19].

#### Process of Providing Education

**Figure 3:**
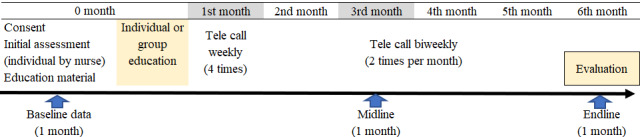
The 6-month program process.

Figure 3 shows the 6-month program process. Because of the pilot study, we set a program for 6 months.

Right after the nurses obtain consent from participants, the nurses conduct the initial assessment using an “Initial assessment sheet” and provide educational materials (“Education booklet” and “Daily monitoring notebook”). If needed, a weight machine, BP machine, medication box, salt measurement spoon, foot care set (monofilament, mirror, nail cutter, nail file, and socks), and eye check chart will be provided. If needed, a blood glucose meter is rented to the patients.

Group education (1 group of less than 20 patients) will be provided at the PHC by the nurses on the scheduled date (1 group education is set for 2 hours).

One week after the group session, telephone calls from the nurses (telenursing education) will be provided weekly on the first month, then biweekly from the second month to the six month. The nurses will contact patients periodically over the telephone, smartphone, or tablet and follow-up on their regular dietary behavior, physical activities, and medicine compliance. They will check clinical examination and laboratory test reports, self-monitoring data, and behavior changes.

In the third month, patients are requested to visit the PHC for regular follow-up by physician and laboratory test. In the sixth month group evaluation for 1 hour and endline data will be collected by the nurses at the PHC.

Patients will start implementing diabetes management right after group education. Each patient will develop monthly goal settings (behavior change plan) related to their diabetes management, daily practice them, and record the results every day in the self-monitoring notebook. The nurses call each patient on the scheduled day, ask about his or her behavior changes and monitor data, then make “step-up goals” for the next month. In addition, based on his or her knowledge and practice level, the nurses provide education using the education booklet. Patients and nurses repeat these activities for 6 months.

### Outcomes and Data Collection Schedule

Since this is a pilot study, we set various indicators (Multimedia Appendix 5). The primary outcome is set as a change in HbA_1c_. As the secondary outcomes, we set various evaluation indicators. The data collection schedule is also set in Multimedia Appendix 5.

Sociodemographic data, clinical history, and lifestyle are taken at the initial assessment (Initial assessment sheet). Satisfaction is measured by the Likert scale (Satisfaction scale). Achievement of monthly goals is measured by percentage. Behavior change is measured by the researcher-developed scale (Lifestyle behavior questionnaire). Self-efficacy is measured by the Diabetes Management Self-Efficacy Scale.

Qualitative data will be interviewed, described, or recorded when it occurs. Engagement and follow-up rate, education material used, complication management, and “patients who have achieved HbA_1c_ in recommended level” will be counted. Cost-effectiveness will be calculated.

### Statistical Analysis

Descriptive statistics will be expressed as frequencies and percentages for categorical variables and mean with SD or median with quantile, distribution, and range for the continuous variables. To compare the categorical variables between pre- and postdata, the chi-square test will be performed. To compare feasibility and efficacy (pre and post), continuous variables will be compared by paired *t* test or one-way ANOVA for the normally distributed and or McNemar test or Wilcoxon signed-rank sum test for the not normally distributed variables after checking the data normality. Qualitative data will be described and categorized if necessary. Statistical significance will be set as <0.05. Data will be analyzed using IBM SPSS Statistics for Windows, Version 25.0 (IBM Corp).

### Ethical Considerations

This study was approved by the institutional review board of the Bangladesh Medical Research Council (BMRC/NREC/2022-2025/336). The researcher will explain the consent procedures including the purpose, procedure, confidentiality, right to withdraw from the study, risk, and benefits, and will obtain written informed consent (ICFs) individually from all the participants. To maintain privacy and confidentiality, data will be collected anonymously. The data will be stored on two password-protected computer sets in the GCCN research room. After deleting all personal identifying information, anonymized data will be used for data analysis, so that research participants will not be identified. To participate in this study, we will not provide any money to the participants. We will provide all the treatments free of cost. As there is no risk or minimum risk to participate in this study, therefore, we will not provide any compassion to participate in this study. We ensure that identifiable features of research participants in any images of the manuscript or supplementary material will not be visible.

#### Interpretation

Data will be cleaned, organized, and verified by investigators as well as checking for any missing data or error data. Then analyze, interpret the findings, conclude, recommend dissemination, and further publication.

#### Training of the Study Nurses

The study nurses received overseas telenursing training on materials development and telenursing services. They have regular oriented sessions about the telenursing research activities with the investigators before the study initiation. They have been receiving protocol-specific hands-on training on how to collect data to perform procedures about self-care and behavioral changes.

#### Procedure for Maintaining Confidentiality

This research will be conducted in accordance with the “Declaration of Helsinki (2013)” by the World Medical Association. This research will be conducted in compliance with the ethical guidelines set forth by the institutional review board of the Bangladesh Medical Research Council (BMRC). The IRB ensures that the human participants are protected through the study. Before enrolling in this study, we will get written informed consent from the participants. Furthermore, we will keep confidentiality maintain through the research process. The researchers of our study will take all necessary steps to ensure the anonymity of the participants. The shared information will not be identified. We will keep all medical information, description of treatment, and results of the laboratory tests confidential, under lock and key, and only our key research staff will have access to this information. The regular monitoring and evaluation system will be conducted to ensure the implementation of preventative measures, or any other potential risk arising during the study. All the study-related information will be kept under lock and key in a secure data store. An appropriate password-based login mechanism would be used to prevent unauthorized access to the server. The collected information would only be visible to authorized research team leaders on a need basis.

No identifiable information would be published anywhere.

#### Reasons for Termination of the Study

The study would be terminated based on the following reasons:

1. Unpredicted ethical concerns that could not be adequately addressed.
2. Unexpected negative events or any harm to participants.
3. Insufficient participant enrollment
4. Financial barriers that could affect the feasibility of the study.

**Discontinuation of Research**

The PI will consider whether to continue the research if any of the following applies:

1. When the ethics review committee has given instructions to change the implementation plan, it is deemed difficult to accept such instructions.
2. When facts or information that undermine the ethical validity or scientific rationality of the research are obtained.
3. When facts or information that undermine the appropriateness of the implementation of the research or the credibility the research results are obtained.

#### Quality Assurance, Control, and Data Monitoring

The study quality will be assured and controlled by the PI and other investigators. Data will be collected by the trained research nurses. The participants’ names will be coded by a unique ID number, and the data will be recorded with the IDs to maintain confidentiality. The data will be cross-checked among the research team. Data will be entered by the nurses. If any discrepancy is found, it will be corrected before data entry. A weekly meeting will be held among the research team to discuss the study and resolve if there will be any study-related confusion.

## Results

The project was funded in 2024. The enrollment of the participants started on October 26, 2024, and as of February 2025, all the participants (N=70) were enrolled. Data analysis will be started after the completion of follow-up data collection and the first results are expected to be submitted for publication in 2026 if all information is available.

## Discussion

### Principal Findings

The results of the research will be disseminated in international scientific conferences and will be published in the *International Science Citation Index (SCI) Journals*. If the results show significance, we will share them with the policymakers, stakeholders, and the research community to implement such interventions in the government and private hospitals. Patients with diabetes will benefit, and it will reduce morbidity and mortality and will reduce patients’ and national economic burden. To control diabetes, health education and lifestyle changes such as food habits, exercise, and medication adherence are imperative. By doing this research, we can improve quality of life by controlling diabetes and preventing complications and death. In this way, we can also decrease the number of hospital visits and admissions and economic burden. This study will increase patients’ health literacy so that they can share their health knowledge with their peers and in the community. The study findings may assist us in the future implementation of telenursing interventions with a large sample size for determining factors that influence the quality of life among patients with diabetes.

### Future Perspectives

By implementing this pilot study, we would like to see the feasibility and significance of this study. We can understand the potential barriers to use the telenursing system. Based on this study, we can revise the methodology and use multiple sites to make further research more effective. If we find our disease management program is effective, we can scale up this program throughout the country. Moreover, government and other nongovernment and private organizations can adopt this program and integrate into the existing health care system of Bangladesh. We can establish a public-private partnership incorporating all the stakeholders to develop a sustainable telenursing system [21].

### Limitations

This study has certain limitations. As we use a purposive sampling technique for selecting the study sites and participants, it causes selection bias. This is a pre- and poststudy design, not using any control group, and thus no causal inferences could be made. We will use appropriate statistics to understand associations in outcome measures to minimize biases [22]. We use 2 rural PHCs from one district; therefore, our findings cannot be generalized for the population of the whole country. Some of the enrolled patients may not understand and follow the study procedures, so we can arrange group education via video conference to minimize this.

### Facilities Available

GCCN has 76 different PHCs throughout Bangladesh. PHC receives patients with diabetes. They also have laboratory facilities to diagnose patients with diabetes. In this study, we have trained investigators and diabetic consultants who will conduct research activities and will provide consultancy. All the data will be entered into a password-protected computer and documents will be kept in the cabinet with a lock and key. Telecommunication will be conducted using smartphones.

### Conclusions

By participating in this study, patients with diabetes will acquire disease-specific self-management skills and can manage a healthy lifestyle and prevent complication.

## Appendix

Multimedia Appendix 1 Education booklet.

Multimedia Appendix 2 Education Booklet_Bengali.

Multimedia Appendix 3 Self-monitoring Book.

Multimedia Appendix 4 Outcome checklist.

Multimedia Appendix 5 The outcome table and data collection schedule.

## Abbreviations

BMRC: Bangladesh Medical Research Council
CGM: continuous glucose monitoring
CKD: chronic kidney disease
FBS: fasting blood sugar
GCCN: Grameen Caledonian College of Nursing
HbA_1c_: hemoglobin A_1c_
ICF: informed consent
LMIC: low- and middle-income country
NCD: noncommunicable disease
PHC: Primary Health Center
PI: principal investigator
RBS: random blood sugar

## Data Availability

The datasets generated and analyzed during this study are available from the corresponding author on reasonable request.

## References

[1] NCD Countdown 2030 collaborators (2018). NCD Countdown 2030: worldwide trends in non-communicable disease mortality and progress towards Sustainable Development Goal target 3.4. Lancet.

[2] (2024). GBD results. Institute for Health Metrics and Evaluation.

[3] Lovic D, Piperidou A, Zografou I, Grassos H, Pittaras A, Manolis A (2020). The growing epidemic of diabetes mellitus. Curr Vasc Pharmacol.

[4] Diabetes. World Health Organization.

[5] Cho NH, Shaw JE, Karuranga S, Huang Y, da Rocha Fernandes JD, Ohlrogge AW, Malanda B (2018). IDF diabetes atlas: global estimates of diabetes prevalence for 2017 and projections for 2045. Diabetes Res Clin Pract.

[6] Farmaki P, Damaskos C, Garmpis N, Garmpi A, Savvanis S, Diamantis E (2021). Complications of the type 2 diabetes mellitus. Curr Cardiol Rev.

[7] National Institutes of Health (NIH). National Institutes of Diabetes and Digestive and Kidney Diseases Managing Diabetes (NIDDK).

[8] Dehghani A, Pourfarid Y, Hojat M (2023). The effect of telenursing education of self-care on health-promoting behaviors in patients with multiple sclerosis during the COVID-19 pandemic: a clinical trial study. Mult Scler Relat Disord.

[9] Disabled world. Definitions of telemedicine fields.

[10] Rao B, Lombardi A (2009). Telemedicine: current status in developed and developing countries. J Drugs Dermatol.

[11] Arnaert A, Delesie L (2001). Telenursing for the elderly. The case for care via video-telephony. J Telemed Telecare.

[12] Caregility. Mitigating the nursing shortage with telenursing.

[13] Mattisson M, Börjeson S, Årestedt K, Lindberg M (2023). Role of interaction for caller satisfaction in telenursing-a cross-sectional survey study. J Clin Nurs.

[14] (2025). Continuous glucose monitors. American Diabetic Association.

[15] (2025). Personalizing health and wellness. IBM.

[16] Hossain MB, Khan MN, Oldroyd JC, Rana J, Magliago DJ, Chowdhury EK, Karim MN, Islam RM (2022). Prevalence of, and risk factors for, diabetes and prediabetes in Bangladesh: evidence from the national survey using a multilevel poisson regression model with a robust variance. PLOS Glob Public Health.

[17] World Health Organization (WHO) 2024. Diabetes Bangladesh 2016 country profile.

[18] Prochaska JO, Velicer WF (1997). The transtheoretical model of health behavior change. Am J Health Promot.

[19] Moriyama M, Kazawa K, Jahan Y, Ikeda M, Mizukawa M, Fukuoka Y, Harada K, Rahman MM (2021). The effectiveness of telenursing for self-management education on cardiometabolic conditions: a pilot project on a remote island of Ōsakikamijima, Japan. J Prim Care Community Health.

[20] Bijl JVD, Poelgeest-Eeltink A V, Shortridge-Baggett L (1999). The psychometric properties of the diabetes management self-efficacy scale for patients with type 2 diabetes mellitus. J Adv Nurs.

[21] Jones A, Bardram JE, Bækgaard Per, Cramer-Petersen CL, Skinner T, Vrangbæk K, Starr L, Nørgaard K, Lind N, Bechmann Christensen M, Glümer C, Wang-Sattler R, Laxy M, Brander E, Heinemann L, Heise T, Schliess F, Ladewig K, Kownatka D (2021). Integrated personalized diabetes management goes Europe: A multi-disciplinary approach to innovating type 2 diabetes care in Europe. Primary Care Diabetes.

[22] Stratton SJ (2019). Quasi-experimental design (Pre-Test and Post-Test Studies) in prehospital and disaster research. Prehosp Disaster Med.

