# Supplementary material for: Telenursing Health Education and Lifestyle Modification Among Patients With Diabetes in Bangladesh: Protocol for a Pilot Study With a Quasi-experimental Pre- and Postintervention Design
Source: JMIR Res Protoc. 2025 May 9;14:e71849. doi: 10.2196/71849 (PMC12102625; doi:10.2196/71849)
Supplement: Multimedia Appendix 1 [file resprot_v14i1e71849_app1.docx]

**What is Diabetes Mellitus?**

Diabetes is a metabolic disorder. When the secretion of insulin from the pancreas decreases or less efficicacious or the pancreas becomes unable to produce insulin, the situation is called diabetes. Diabetes is a disease that can go unnoticed and return without any symptoms.

**Types of Diabetes Mellitus**

- Type I ( Insulin dependant diabetes mellitus)
  - Generally occurs at the age of 10-12 years of age
- Type II ( Non-insulin dependant diabetes mellitus)
  - Generally occurs at the age of over 30 years but due to sedentary lifestyle now-a-days the people below 30 years are developing diabetes.
- Gestational Diabetes
  - It occurs during pregnancy

**Mechanism of diabetes**

If left untreated, blood vessels throughout the body will fall apart. Let's notice this as soon as possible and take action.

**In Non-diabetic subject-**

1. When you eat or drink, the carbohydrates (carbohydrates) are broken down into glucose and transported into the bloodstream (=blood sugar).
2. When the amount of glucose in the blood increases, the pancreas secretes the extra amount of “insulin,” which lowers blood sugar levels.
3. Insulin works to take glucose into cells (liver, muscle, fat cells).
4. The glucose that is taken into cells is used as energy. Blood sugar levels are kept within a certain range.

“**Insulin is the only hormone that can lower blood sugar levels. Only when insulin takes glucose into the cells is it used for energy.''**

**In Diabetic person, when blood glucose is consistently high-**

**Pancreas:** I'm already... tired...!

The pancreas becomes tired as it tries to produce a lot of insulin.

**Insulin:** Huh? It's getting harder to take in.

Insulins are too busy to take glucose from the bloodstream.

**Glucose:** I don't have enough insulin

There is not enough insulin to transport glucose into cells

- And then insufficient insulin secretion as the pancreas is too tired to produce continuous insulin.
- Even when insulin tries to take glucose into cells, it is difficult to take it in because of less potency of insulin.
- High insulin resistance
- “It resists the action of insulin, making it less effective.”
- “Insulin becomes difficult to produce or becomes insufficient.”

**“Glucose increases in the blood → becomes hyperglycemic → hyperglycemia continues →**

**diabetes develops**.

**Note:** Because of insulin deficiency, protein, and fat metabolism also becomes inappropriate, as the extra carbohydrates are transformed into glycogen and triglycerides and accumulate in the muscles and fat cells.

**Basics of treatment for diabetes**

**Diet adjustment:**

● Appropriate energy (calorie) intake

● Adequate protein intake

**You have to……**

1. Pay attention to the order in which you eat

Vegetables (dietary fiber) Meat and fish (protein) Rice (staple food)

2. Eat slowly and chew thoroughly

3. Eating while eating and not eating inadvertently (carelessly).

**Exercise therapy:**

- Stretching
- Muscle training
- Aerobic exercise.

**You have to…………**

1. Move frequently, walk frequently

2. Use the stairs rather than the elevator

3. Move a little after eating

4. Do not sit for long time

**Drug therapy:**

- Know how to take medicines, their effects and dose adjustment if requires.
- Hypoglycemia response
- How to deal with sick days

You have to ………….

1. Take a fixed amount while you drink
2. Let’s drink at a fixed time

**Do not forget!**

- Self-monitoring of blood glucose
- Foot Care
- No Smoking
- Regular follow-up

**Lifestyles to maintain blood sugar level…………………….**

**The Dietary Instructions:**

**Daily energy (calorie) intake:** standard body weight x calorie factor

Standard body weight= Height in cm-100

The calorie factor varies from 20 to 45 according to the BMI and physical activity level.

Restrict protein intake from stage 3 diabetic nephropathy.

**Please check with your nephrologist and consult with a registered dietitian who specializes in kidneys.**

Salt intake is important regardless of the stage of the disease. [5g or less per day]

Diabetic people's calorie calculation per day:

Diabetic people with nephropathy calorie calculation per day:

**How to prepare your daily meal…??**

3 main meals and 2-3 snacks in a day

First, let’s prepare the three plates

- Mainly vegetable
- Standard foods such as rice or bread or ruti
- Meat, fish, or eggs

**Eat a variety of foods in a well-balanced manner**

Recommended amount of meat, fish, and eggs to eat per day (The amount of meat or fish that fits in your palm).


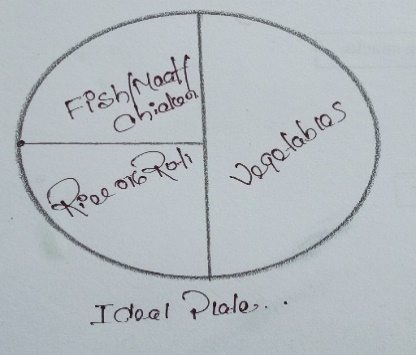
**1 g of oil = 9 kcal**

**All types of oil have the same calories.**

**All types of oil/ghee= 3 teaspoon= 135 Calories**

Vegetables: 350 g per day.

Light-colored vegetables: green and yellow vegetables

**You can eat following the order of diet to maintain your 1600 Kcal per day….**

3 Ruti=225 calories

1 cup Dal= 100 calorie

1 boiled egg= 75 calorie

Coocked Vegetables as wish

Total=400 calories

Morning

1 cup chira=150 calorie

Mid-morning snacks

2 cups of rice= 300 calorie

1 piece of row fish= 40 calorie

1 cup cooked dal= 100 calorie

Cooked Vegetables as wish.

Total=440calories

Lunch

**৪ টি ক্রেকার Biscuit= 75 Calories**

1 cup of sugar-free tea

Total=75 calorie

Evening snacks

4 Ruti= 300 calories

1 cup cooked Dal= 100 calorie

Total=400 calories

Dinner

You can use the following utensils for measurement:

1 cup= 150 ml=120 gm

1 glass 250 ml= 240 gm

1 teaspoon= 5 g

1 tablespoon= 15g

1 bowl= 200 ml

**How to eat without rapidly raising blood sugar levels (3 techniques)**

**1 Eat vegetables first and carbohydrates last**

Carbohydrates, which are found in large amounts in foods such as rice, bread, and noodles, raise blood sugar levels after meals. Carbohydrates include sugar and dietary fiber, but it is sugar that raises blood sugar levels. Vegetables can be eaten not only raw vegetables such as salads but also cooked ones or those containing meat or fish.

**2 Eat slowly**

Eating quickly causes blood sugar levels to rise rapidly. Additionally, you may not feel full and tend to overeat. Enjoy your meals with your eyes and enjoy them slowly.

**3 Chew your food thoroughly**

If you chew a lot, your blood sugar level will rise slowly. It stimulates the satiety center, prevents overeating, and improves blood flow to the brain.

**Fluctuations in blood sugar levels by nutrients**

**Not only sugar but also other nutrients help to raise blood sugar levels.**

Carbohydrates raise blood sugar levels by 100% after eating.

Protein raises blood sugar levels by 50% after a short delay after eating.

In fact, after eating fat, blood sugar levels slowly rise over time.

If your fasting blood sugar level is high in the morning even though you haven't eaten anything particularly sweet or sugar, you may have eaten something greasy or fatty the night before.


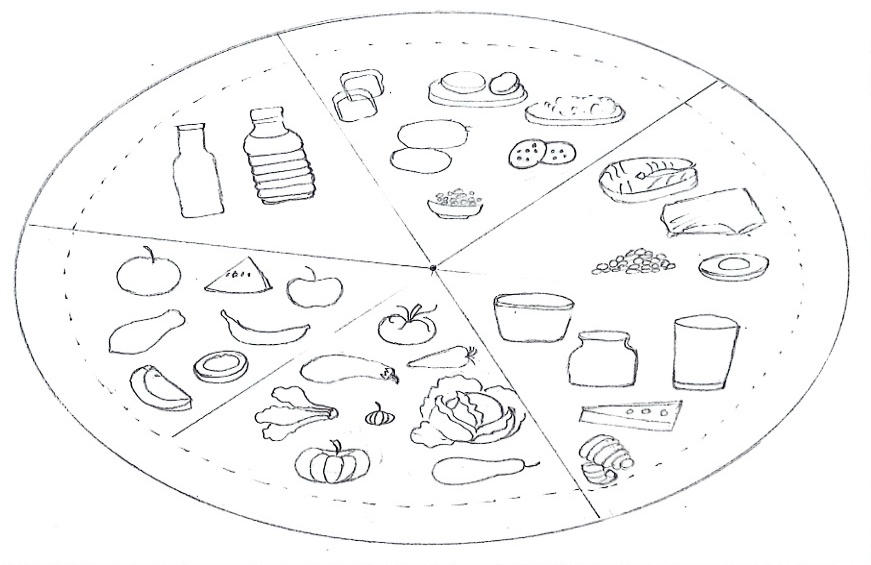


Food containing carbohydrates, proteins, fats

**What to do during Ramadan?**

**At least 2 months before Ramadan consult with your doctor.**

Medicines schedule

Morning dose: During Iftar

Night dose: During Sheri (make it half)

Break your fasting; if your blood glucose level is less than 3.9 or more than 16.7.

**What and how you will eat during Ramadan**

**Iftar:** Sherbet; (Isbgul, tokma, tetul, lemon, raw mango juice), Fresh fruit juice without sugar

Take a meal similar to breakfast

**Dinner:** Meal equal to dinner.

**Sheri:** Take a meal at the end time of Sheri. Meal equal to lunch.

You can take either rice and fruits or rice and milk together but not rice, fruits, and milk at a single meal.

**NOTE:** During Ramadan do not need to do physical exercise. Just follow your daily works.

Those who have no complications with the kidney, heart, and nervous system as well as who have normal blood pressure levels can perform all kinds of work.

**Tips to improve exercise effectiveness**

- Fixed time of exercise daily.
- Do Similar exercise

Daily 30 minutes for at least 5 days in a week.

- At least 1 hour after main meals

Blood sugar levels can go down If you exercise after having a meal.

Blood sugar levels reach their highest 1 to 1.5 hours after a meal.

★However, if you experience a spike in blood sugar levels, it may be helpful to take a short walk immediately after eating.

A blood sugar spike is a sudden increase in blood sugar after a meal. It is a state where there are rises and falls.

Consume 200-300kcal per day through exercise

Aim to consume about 15% of your daily calorie intake

If you no experience of exercise then build up the exercise time.

Goal of physical exercise;

150 minutes of moderate-intensity physical activity per week

e.g.; Brisk walking for around 30 minutes for 5 days a week.

**Those who are not able to manage time 5 days a week can exercise, more than 3 times a week**

(The effect of lowering blood sugar levels lasts for about 2 days after exercise)

a… If you exercise for one day, the effect of lowering blood sugar levels will last for about two days, so if you exercise every two days, the effect will continue to be high and you can keep your blood sugar levels in good condition.

b... If there are many days when you do not exercise, and the intervals between days when you exercise become long, the effect of lowering blood sugar levels will be diminished.

Note: Do not do exercise in barefoot and eat enough water during exercise.

[How to check BMI (degree of obesity)] **BMI=Weight/Height^2^ (kg/m^2^**)

Underweight <18.5 kg/m2

Normal Weight 18.5 to 22.9 kg/m2

Overweight 23- 26.9 kg/m2

Obese > 27 kg/m2

Severely Obese 40 and above

**Asian cutoff**

**Exercise in a well-balanced manner**

If you feel like it’s fun, you should be able to breathe a little and sweat a little. You should not go so far as to make you feel very tired and sweaty………... jogging, walking or swimming**.**

1. **warming up** (5 to 10 minutes)

Try to do exercise same time in a day……….

Be sure to do this to prevent accidents and breakdowns during exercise.

Ex. A simple walk is just enough to warm your body slightly

1. **Muscle Stretching exercise** (5-10 minutes)

Stretch (expand) your muscles slowly without building momentum.

- Stretching to prevent back pain, etc.

Prolonged sitting should be interrupted every 30 minutes.

1. **Strength Training exercise** ( 30 minutes)

Do this until you are slightly out of breath and slightly sweaty.

・Sit on a chair and continue raising and lowering..

- Going up and down stairs, etc

1. **cool down** (5-10 minutes)

This is done to prevent accidents and muscle pain after exercise.

Exercise with a light load until your breathing calms down.

**Do not do exercise if…………………………………………….**

- Blood sugar level is higher than usual, >16.7mmol/l

If you have complications such as angina pectoris, myocardial infarction, or diabetic nephropathy, consult with your doctor about the intensity, duration, and heart rate of your exercise.

- Blood pressure is higher than usual >160/100 mm of Hg
- Pulse is faster than usual
- I caught a cold/I don't feel well
- Headache, abdominal pain, diarrhea, and fever
- Hangover/Lack of sleep
- Feeling unusually tired
- Feeling severe pain in joints and muscles
- Any acute illness

Stop exercising if you experience chest pain.

If you experience pain, stop exercising and sit or lie down to rest. If the pain does not subside even after a short rest, go to a medical institution immediately. Pain may appear not only in the chest area, but also over a wide area from the chest to (both) shoulders and arms, or from the chest to the waist and back.

**Note 1**: Those who have neuropathy or osteoarthritis in the back or knee no walking, jogging, or treadmill. For them swimming, and cycling are recommended.

For any who have retinopathy swimming, walking, or cycling are recommended. No weight lifting.

Exercise and special situations………..

- Retinopathy
- Foot ulcer
- Pregnancy

**Note 2**: Those who work very hard (ex. players and laborers) do not need to do extra exercise.

**Foot care**

Foot care is important to ensure your health. Foot is the neglected part of the body. If foots are not taken care properly, both neuropathy and peripheral vascular disease affect foot.

There is a high possibility of diabetic neuropathy, if

- Loss of sensation
- Abnormal sensation (Feels like walking on stroke)
- Burning, tingling, pin and needle sensation

Daily foot care………………………………..

1. Wash your feet with lukewarm water

Before pouring hot water on your feet, check the temperature with your hand to make sure it's not too hot. Do not forget to wash between your toes.

1. Wipe with a dry and soft towel

Don't forget to wipe between your toes!

1. Apply lotion

Choose one that is not sticky but do not apply it between your toes!

**4**. Carefully observe the condition of your skin

□ Skin color, skin texture □ swelling □ Cracks and scratches □ Ingrown toenails and nail color

□ Are the ingrown toenails white and thick? □Blister, □ Shoe blister, □Dryness □ Stiffness

5. Always wear clean socks with shoes.

Immediately when a wound occurs

Before you put them on, make sure nothing is inside your shoes.

Make sure the socks are not wet.

Five-finger socks are recommended

1. Do not walk barefoot

Always use sandals while walking to avoid scratches/ injuries. Always pay attention to your feet daily.

**Treatment for blood sugar control**

The higher the number, the more poorly controlled your blood sugar is and complications are more likely to occur

The recommended HbA1c level according to situation:

| The goal | Goals 1  Aiming to normalize blood sugar | Goal 2  Preventing complications | Goals 3  Treatment intensification is difficult or stringent; in the elderly with multiple co-morbidities. |
| --- | --- | --- | --- |
| HbA1c (%) | Less than 6.5 | Less than 7.0 | 7.8% |

| Preconception | <6.5% |
| --- | --- |
| Non-pregnant women | <7% |
| For children, elderly and with co-morbidity more relaxed | |

HbA1c tells you your average blood sugar level over the past 3 months.

Blood Sugar Level (mmol/l) = mg/dl % 18

| Estimated fasting blood sugar level | less than 7.0 mmol/l |
| --- | --- |
| Estimated blood sugar level for 2 hours after meals | less than 10 mmol/l |

**To prevent diabetic complications the following things should be controlled along with blood glucose level**.

So, The person who does not have raised complications yet has to follow

| Triglycerides | Less than 150 mg/dl |
| --- | --- |
| HDL | Mae: more than 40 mg/dl  Female: more than 50 mg/dl |
| LDL | Less than 100 mg/dl ℮ cardiac event < 70 mg/dl |
| BMI | < 23 kg/m2 |
| Blood Pressure | < 130/80 mm of Hg |

| HbA1c is glucose bound to hemoglobin contained in red blood cells in the blood. Red blood cells circulate through the blood while binding to glucose in the blood. The more glucose in the blood, the higher the HbA1c value. |
| --- |

**Note:** Even if the HbA1c is the same for 2 different people blood sugar levels throughout the day are different, so it means that those who have more blood sugar levels fluctuation throughout the day are more prone to risk of blockage of large blood vessels.

Mr. A and Mr. B

HbA1c 7% is the same but different blood sugar level

**It is necessary to self-monitoring blood sugar levels along with HbA1c**

| **Note 1.** A goal that can be achieved only with appropriate diet and exercise therapy, or even with drug therapy without side effects such as hypoglycemia.  **Note 2.** From the perspective of preventing complications, set the target HbA1c value to less than 7%. The corresponding blood sugar level is a rough guideline a fasting blood sugar level of less than 7.0 mmol/l and a 2-hour postprandial blood sugar level of less than 10 mmol/l.  **Note 3.** Used as a goal when it is difficult to strengthen treatment due to side effects such as hypoglycemia or other reasons.  **Note 4.** All target values are for adults, and pregnant cases are excluded.  *From the Japan Diabetes Society Diabetes Treatment Guide 2016-2017* |
| --- |

Feeling sleepy after eating? This could be a sign of danger

**Sudden hyperglycemia only after meals = blood sugar spike**

This is because glucose cannot be taken into cells properly and the body secretes a large amount of insulin to lower the blood sugar levels.

“Blood sugar spikes” are fluctuations in blood sugar levels in which postprandial blood sugar levels rise and fall rapidly in a short period compared to the hourly blood sugar levels.

People with postprandial hyperglycemia; Blood sugarrises to a high peak after every meal.

Sleep at least 2-3 hours after and do not try to go to bedjust after having a meal.

Healthy person: Blood sugar stays normal.

Repeated spikes in blood sugar levels progress to atherosclerosis, which eventually leads to myocardial infarction and increases the risk of developing cancer.

Hypoglycemia occurs when the blood glucose level is less than 3.9 mmol/l.

**Symptoms of Hypoglycemia**

| **Blood sugar level** | **Symptoms**  Blurred vision, unusual hunger, weakness, fatigue, difficulty concentrating, cold sweats, palpitations, tremors, pale and red face, slurred speech  Loss of consciousness, abnormal behavior, convulsion, coma. |
| --- | --- |
| Level 1: <3.9 to 3 mmol/l Can be managed by own. |  |
| Level 2: < 3 mmol/l Can be managed by own |  |
| Level 3: < 3.9 mmol/l |  |

Recurrent hypoglycemia consequences behavioral changes and cognitive impairments and life-threatening cardiovascular disease.

**Dealing with hypoglycemia (at home) ………………………………………………………………………………………………….**

**When conscious:**

- 15 g carbohydrate means take 3 tsf glucose or food equal to one ruti (rice, biscuit or bread). Then wait for 15 minutes. Then check blood glucose level which should be a state limit of 5.5 mmol/l. If not recovered then,
- 3-4 teaspoons of sugar (1 teaspoon 4 gm sugar) or honey 3 teaspoon. Juice containing sugar (glucose) half glass is also fine.

These measures are usually adequate to raise blood glucose to a reasonably safe limit (5.5 mmol/l).

- Take repeated food/drink every 15 minutes and check your blood sugar level after 15 minutes, and if it hasn't improved, take the same amount of glucose or sugar again.

**Note:** Do not exercise after taking insulin or hypoglycemic drugs.

**Note 1:** People who are taking oran antidiabetic drugs must always take glucose.

**When unconscious:** (Tell people around you to do this when you are in a hypoglycemic condition)

Rub sugar, or glucose, on your gums, inside your lips, and on your tongue. At the same time, take him to a medical facility or call an ambulance.

Do not put liquids into the mouth if the person is unconscious or drowsy. It may enter the airway and cause suffocation.

**How to deal with sick days (days when you feel unwell) such as fever, vomiting and diarrhoea**

During sick days it is very difficult to keep the blood sugar level in control. Cold, fever, and vomiting can cause severe illness. You have to remember during sick days …………………………

Fever (Temperature > 100 degrees F or 38 ℃), vomiting, and diarrhea often cause hyperglycemia and sometimes hypoglycemia.

Another symptom is that you cannot eat or cannot eat much.

**If any of these apply to you, contact your doctor.**

**How can I manage this situation?.......................................................................................................................**

- Check blood sugar every 4 hours. (write the measurement in the self-monitoring book). The target is, to keep the blood sugar level between 7-10 mmol/l.
- Please take frequent small meals.
- During diarrhea, you can have very rice water.
- During hypoglycemia sweetened juice and hyperglycemia just water or calorie-free drink.

Water-120-180 ml in each half-hourly.

- Stop oral drugs (e.g.: Metformin) temporarily during vomiting and diarrhea.
- Do not stop taking insulin but take the adjusted dose.

Note: For making any decision regarding medications you need to contact your doctor.

- Physical exercise postponed during sick days.
- When you don't have much to eat, if possible, eat easily digestible foods and drinks that contain sugar and electrolytes.
- Eat as normal as possible.

Follow these until blood glucose is < 12 mmol/l.

- **If you cannot contact your doctor, such as at night, please visit the emergency department.**

**You need to go to hospital;**

- Vomiting or diarrhoea persisting for more than 6 hours

**You can contact your telehealth nurses for primary care**

- Blood glucose level is more than 16.7 mmol/l.
- If blood glucose level remains at 13.5 mmol/l for more than 24 hours.
- If temperature remains 100 degrees Fahrenheit for more than 24 hours.
- You have been sick for 3 days and have not recovered
- Abdominal pain
- Breathing difficulties and excessive dryness of lips and tongue.
- Feeling Drowsy
- Who has kidney or heart disease
- When Blood glucose level becomes lower to 3.9 mmol/l or 70 mg/dl.

**Stages of Diabetic Nephropathy:**

| Stages | 1 | 2 | 3 | 4 | 5 |
| --- | --- | --- | --- | --- | --- |
| GFR (eGFR) (ml/min/1.73 m^2^) =Kidney function | 30 or more | | | less than 30 | |
| Urinary albumin  value (mg/gCr) | Normal albuminuria <30 | Microalbuminuria 30-299 | Overt  albuminuria 300 or more | (Regardless of the presence or absence of urine protein) | |
| Urine protein value (g/gCr) |  |  | persistent urine protein 0.5 or more (1+) |  |  |
| Kidney function status | Early stage of nephropathy | early nephropathy stage | Obvious nephropathy stage | renal failure stage | Dialysis treatment period |
| **Treatments**  **Tergets** | Salt 5g/day |  |  |  |  |
|  | "Energy intake: 25~30kcal/kg/day (in case of obesity: 25kcal/kg/day is also possible) | |  |  |  |
|  |  |  | "Protein intake. 0.8~1.0g/kg/day | "Protein intake 0.6~0.8g/kg/day |  |
|  | BMI less than 25 |  |  |  |  |
|  | Exercise therapy |  | consult a doctor to see if there are any heart problems |  |  |
|  | Blood sugar control HbA1c less than 7.0% | |  |  |  |
|  | Blood pressure control less than 130/80 mmHg | |  |  |  |
|  | Lipid management LDL cholesterol less than 120 mg/dl, less than 100mg/dl if heart complications | | |  |  |

Prepared based on the Japanese Diabetes Society Diabetes Treatment Guide 2016-2017, published by Bunkodo, 2016 (P82-85) Japan Diabetes Society website, Report of the Diabetic Joint Committee (Revision of diabetic properties)

**Residual kidney function determined by blood test results**

[Example of test data received at a medical institution for a 77-year-old patient]

| Serum creatinine | 1.0 mg/dl |
| --- | --- |
| eGFR | 53.76 ml/min/1.73 m^2^ |
| Serum Urea | 28.38 mg/dl |
| Serum Uric Acid | 4.80 mg/dl |

[Example of test data received at a medical institution for a 52-year-old patient]

| Serum creatinine | 2.64 mg/dl |
| --- | --- |
| eGFR | 18.99 ml/min/1.73 m^2^ |
| Serum Urea | 59.39 mg/dl |
| Serum Uric Acid | 8.10 mg/dl |

**We can see that when serum creatinine level raised the kidney function has been dropedd.**

When Kidney function is 10%, and dialysis is often required.

**A reference of kidney function test’s value**

| **Test Name** | **Normal Range** |
| --- | --- |
| Serum Creatinine | **0.55-1.3 mg/dl** |
| Potassium | **3.5-5.2 mEq/l** |
| Sodium | **136-148 mEq/l** |
| Chloride | **98-108 mEq/l** |
| Calcium | **8.5-10.03 mg/dl** |
| eGFR | **>60 ml/min/**1.73 m^2^ |

If you have diabetes, kidney disease, or high blood pressure, you are at increased risk of end-stage renal failure (starting dialysis treatment, or just before transitioning to dialysis treatment), stroke, myocardial infarction, and ultimate death.

**Know your risk of diabetes complications……**

Where do complications of diabetes occur?

| Large blood vessel disorders |
| --- |
| Life-threatening complications |

| Small blood vessel disorders |
| --- |
| Complications that interfere with daily life |

| Stroke |
| --- |

Needed; Once a year: Carotid artery ultrasound examination

People with high blood pressure should be cautious.

| Diabetic retinopathy |
| --- |

Blindness if worsened

Needed; to visit your doctor regularly

Once every 6 months to a year: Fundus examination

| Heart Attack |
| --- |

Especially obese people (BMI ≥25) should be careful.

Needed; Once a year: ECG test

| Diabetic nephropathy |
| --- |

If it worsens, dialysis and death from heart failure

Peripheral Vascular Disease

Blood test at the time of visit to hospital

Diabetic neuropathy

Clogged or blocked blood becomes difficult to flow to the body.

If the situation worsens, lower limb amputation may be required.

As diabetes progresses, dementia and bedridded care are required.

Observe the soles of your feet, nails, etc. daily

**Underweight <18.5**

**Normal Weight 18.5 to 23.0**

**Moderate risk/overweight 23 to <27.5**

**High risk/Obese _>27.5**

**Underweight <18.5**

**Normal Weight 18.5 to 23.0**

**Moderate risk/overweight 23 to <27.5**

**High risk/Obese _>27.5**

**Antidiabetic drugs:**

Type of medicine: Main action:

**Sulfonylurea (SU) drugs: (Glipizide, gliclazide, glimepiride, glibenclamide):** Act directly on the pancreas to stimulate insulin secretion from beta cells.

**Non-sulfonylureas: (Repaglinide, Nateglinide):** It also stimulates insulin secretions from the pancreas.

**Biguanide drugs:** **(Metformin):** (Reduce insulin resistance). Suppresses new glucose production in the liver

**Thiazolidinediones: (Pioglitazone, rosiglitazone):** Improves insulin sensitivity in skeletal muscle and liver, making insulin more effective and lowering blood sugar.

**Alpha-glucosidase inhibitor (∞-G1):** **(Acarbose, miglitol, voglibose)** Suppresses the function of digestive enzymes and delays sugar absorption. Take this medicine just before meals so that the food and medicine mix to produce the effect.

**DPP-4 inhibitor: (Linagliptin, alogliptin, sitagliptin, etc.):** Promotes insulin secretion according to blood sugar level. It suppresses the secretion of glucagon, a hormone that increases blood sugar levels.

**SGLT2 inhibitor:** **(Empagliflozin, etc.):** Inhibits sugar reabsorption in the kidneys, promotes glucose excretion into the urine, and lowers blood sugar levels.

**Rapid-acting insulin secretion promoter (glinide drug):** Causes insulin to be secreted quickly after meals. The effect is short-lived, postprandial hyperglycemia improvement.

**Insulin:**

The rapid-acting insulins are….

- Insulin Lispro
- Insulin Faster Aspart
- Insulin Aspart
- Insulin Glulisine

Long-acting insulin analogue

- Insulin Detemir
- Insulin Glarine
- Insulin Degludec

Premixed Insulin (30/70, 50/50, 25/75)

- Biphasic Human Insulin
- Biphasic Insulin Aspart
- Biphasic Insulin Lispro

**Reference:** National Guideline on Diabetes Mellitus; Non-communicable Disease Control Program, Directorate General of Health Services, Ministry of Health and Family Welfare, First edition, August, 2023.

**Three promises of diabetes medicine**

**At first, know about your medicines and doses and timing.**

**1. Never forget to take medicine**

It is strictly forbidden to forget to take your medicine, but if you frequently forget to take it, consult your doctor and ask them to change the dosage to suit your lifestyle. You may be able to change the type of medicine or take it less often.

**3 ways to remember to take medicine**

- Put the medicines in one container and place it in a visible place.
- Set an alarm on your phone.
- Mark notes, notebooks, and calendars.
- You can tell one of your family members to remind you to take regular medicines.

1. **Observe the timing (time) of taking medicines as per doctor’s advice**

Dosage time:

- Take 30 minutes before having a meal.
- Take 30 minutes after meals
- Take between meals=2 hours after meals

**3. Ensur regular follow-up without any gap between treatment.**
