# Supplementary material for: Telenursing Health Education and Lifestyle Modification Among Patients With Diabetes in Bangladesh: Protocol for a Pilot Study With a Quasi-experimental Pre- and Postintervention Design
Source: JMIR Res Protoc. 2025 May 9;14:e71849. doi: 10.2196/71849 (PMC12102625; doi:10.2196/71849)
Supplement: Multimedia Appendix 2 [file resprot_v14i1e71849_app2.pdf]

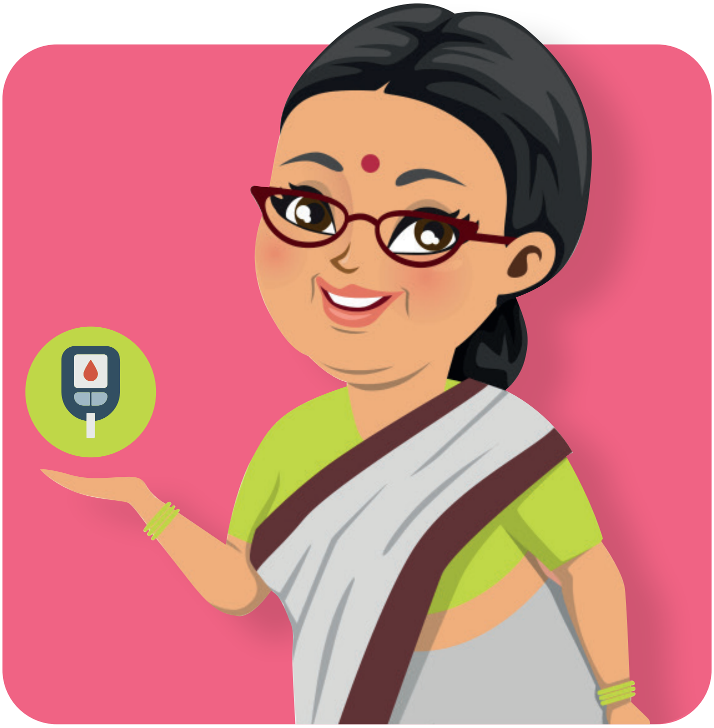

ডায়াবেটিস নিয়েও হাসিখুশি জীবনযাপন করা সম্ভব যখন

HbA1c নিয়ন্ত্রণে থাকবে।

ডায়াবেটিসের জটিলতাগুলো কমিয়ে আনা সম্ভব হবে।

ডায়াবেটিস সম্পর্কে সচেতনতা বৃদ্ধি পাবে।

পাঠ্য লেখনী এবং অনুবাদ

১. লুসি মন্ডল
আর.এন., বি.এস.এন.
টেলিনার্স সার্ভিস অফিসার, গ্রামীণ ক্যালেডোনিয়ান কলেজ অব নার্সিং।

২.আখি রায় মিতা
আর.এন., বি.এস.এন,
টেলিনার্স সার্ভিস অফিসার, গ্রামীণ ক্যালেডোনিয়ান কলেজ অব নার্সিং।

বই ডিজাইন

মিস্টার পল মন্ডল, সিনিয়র ভিজুয়ালাইজার।

সাধারণ সুপারভাইজার

১. মিচিকো মরিয়ামা
আর.এন., এম.এস.এন., পি.এইচ.ডি.
প্রফেসর, ডিপার্টমেন্ট অব অ্যাডাল্ট নার্সিং ডেভেলপমেন্ট,
ডিপার্টমেন্ট অব অ্যাপ্লাইড লাইফ সায়েন্সেস, গ্রাজুয়েট স্কুল অব বায়োমেডিকেল
এন্ড হেলথ্ সায়েন্সেস, হিরোশিমা ইউনিভার্সিটি, জাপান।

২. কে.এ.টি.এম. এহ্‌সানুল হক
এম.বি.বি.এস., ডি.টি.এম. এম.এস.সি, পি.এইচ.ডি,
গ্রাজুয়েট স্কুল অব বায়োমেডিকেল এন্ড হেলথ্ সায়েন্সেস,
হিরোশিমা ইউনিভার্সিটি, জাপান।

মেডিকেল সুপারভাইজার

ডাক্তার ফারিয়া আফসানা
সহযোগী অধ্যাপক এবং ইউনিট-২ প্রধান, এন্ডোক্রাইনোলোজি বিভাগ,
বারডেম জেনারেল হাসপাতাল এবং আই.এম.সি।

প্রাতিষ্ঠানিক সুপারভাইজার

নিরু শামসুন নাহার
এম.এস.সি, বি.এস.সি, ডিপ (এ.এন.),
ডিপ (এন.), ডিপ (এম),
অধ্যক্ষ, গ্রামীন ক্যালেডোনিয়ান কলেজ অব নার্সিং।

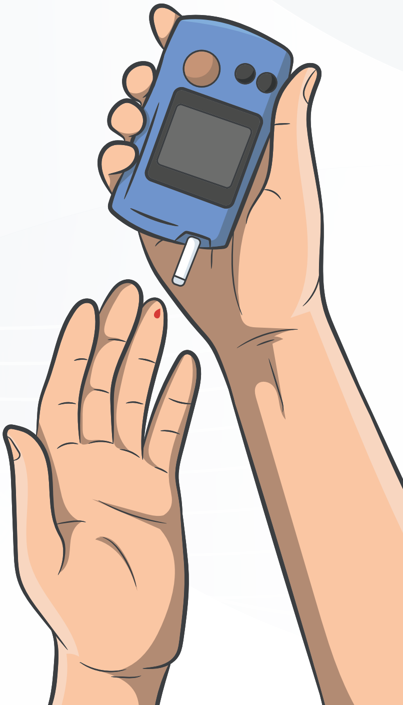

সূচিপত্র

ডায়াবেটিস সম্পর্কে জানুন

ডায়াবেটিস এবং ডায়াবেটিসের প্রকারভেদ (ধরণ-১ এবং ধরণ-২) ৩

ডায়াবেটিস কিভাবে হয়...৪

ডায়াবেটিসের মৌলিক চিকিৎসাসমূহ...৬

সঠিক বি.এম.আই. (BMI) এবং দৈনন্দিন কতটুকু ক্যালরি/শক্তি প্রয়োজন সে সম্পর্কে জানুন

বি.এম.আই (BMI) -এর সাধারণ মাত্রা এবং কিভাবে বি.এম.আই. গণনা করা যায়....................৭

ডায়েট/খাবার সম্পর্কে জানুন

সুষম খাদ্য (শাকসবজি, কার্বোহাইড্রেট/শর্করা, প্রোটিন/আমিষ, ফ্যাট/চর্বি এবং খনিজ).................৮

দৈনন্দিন খাবারের নির্দেশনা...৯

রক্তে গ্লুকোজের মাত্রা খুব দ্রুত বাড়তে না দেওয়ার ৩টি উপায়..১০

রমজান মাসে যে-সকল নিয়ম মেনে চলতে হবে...১১

ব্যায়াম সম্পর্কে জানুন

ব্যায়ামের কার্যকারিতা বাড়ানোর উপায়..১২

ব্যায়ামের ধাপসমূহ...১৩

যে সকল অবস্থাতে ব্যায়াম এড়িয়ে চলতে হবে..১৪

পায়ের যত্ন

দিনে কতবার এবং কিভাবে পায়ের যত্ন নিতে হবে...১৫

HbA1c এবং হাইপোগ্লাইসেমিয়া সম্পর্কে জানুন

HbA1c কী এবং রক্তে HbA1c এবং গ্লুকোজের-এর লক্ষ্যমাত্রা..১৬

হাইপোগ্লাইসেমিয়ার লক্ষণগুলো সনাক্তকরণের পাশাপাশি প্রয়োজনীয় ব্যবস্থাপনা.........................১৭

অসুস্থতার দিনগুলোতে করণীয়..১৯

ডায়াবেটিসের জটিলতাসমূহ

ডায়াবেটিসের জটিলতা (যেমন; চোখের সমস্যা, পায়ের ক্ষত, কিডনিজনিত সমস্যা এবং হার্টের সমস্যা) এবং জটিলতা নিয়ন্ত্রণে চিকিৎসা...২০

ডায়াবেটিক নেফ্রোপ্যাথির ধাপসমূহ ও চিকিৎসা...২১

রক্ত পরীক্ষার ফলাফল থেকে কিডনির কার্যক্ষমতা জানুন...২২

অ্যান্টিডায়াবেটিক ড্রাগসমূহ/ঔষধ ২৩

ঔষধ খাওয়ার ৩টি নিয়মাবলী...২৪

ডায়াবেটিস সম্পর্কে জানুন

ডায়াবেটিসের পূর্ণ নাম ডায়াবেটিস মেলাইটাস যাকে বাংলাতে “বহুমূত্র” রোগ বলা হয়। ডায়াবেটিস একটি বিপাকজনিত ব্যাধি যেখানে রক্তে গ্লুকোজের মাত্রা নিয়ন্ত্রণহীনভাবে বাড়তে থাকে। যখন অগ্ন্যাশয় থেকে ইনসুলিন নামক হরমোন তৈরী হওয়া কমে যায় বা অগ্ন্যাশয় কোনো কারণে পর্যাপ্ত ইনসুলিন তৈরি করতে অক্ষম হয়ে পড়ে অথবা অগ্ন্যাশয় থেকে একেবারেই ইনসুলিন তৈরী হওয়া বন্ধ হয়ে যায় তখন সেই অবস্থাকে ডায়াবেটিস বলে। ডায়াবেটিস এমন একটি রোগ যা উল্লেখযোগ্য কোনো লক্ষণ ছাড়াই শরীরে বেড়ে উঠতে পারে।

ডায়াবেটিসের প্রকারভেদ

(১) ধরণ-১ (Type 1): ইনসুলিন নির্ভর ডায়াবেটিস মেলাইটাস

এই ধরণের ডায়াবেটিস সাধারণত কম বয়সীদের মধ্যে হয়ে থাকে।

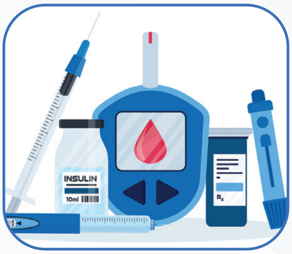

(২) ধরণ-২ (Type 2): নন-ইনসুলিন নির্ভর ডায়াবেটিস মেলাইটাস

এই ধরণের ডায়াবেটিস সাধারণত ৩০ বছর বয়সের পরে হয়ে থাকে। কিন্তু বর্তমানে যারা আরামপ্রিয় জীবনযাপনে অভ্যস্ত তাদের মধ্যে ৩০ বছরের আগেই এ ধরণের ডায়াবেটিসে আক্রান্ত হওয়ার সংখ্যা দিন দিন বেড়ে চলেছে।

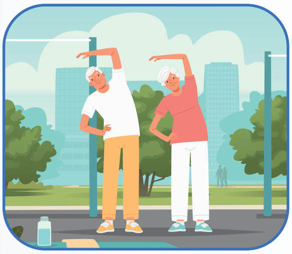

(৩) গর্ভকালীন ডায়াবেটিস

অনেকসময় গর্ভাবস্থায় মায়েদের মধ্যে ডায়াবেটিস ধরা পড়ে। প্রসবের পর আর ডায়াবেটিস থাকে না।

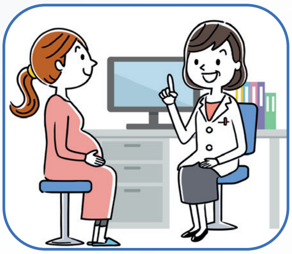

যদি সময়মত ডায়াবেটিসের চিকিৎসা শুরু করা না হয় তাহলে একসময় সারা শরীরের রক্তনালীগুলো ভেঙে পড়ে।

ডায়াবেটিস কিভাবে হয়?

১ যখন আমরা কোনো খাবার খাই তখন খাবারের কার্বোহাইড্রেট/শর্করা ভেঙে গ্লুকোজে পরিণত হয় এবং সেই গ্লুকোজ শরীরের রক্ত প্রবাহে মিশে যায় (একে ব্লাড সুগার বলা হয়)।

২ যখন রক্তে গ্লুকোজের পরিমাণ বেড়ে যায়, তখন অগ্ন্যাশয় "ইনসুলিন" নামক হরমোন নিঃসৃত করে। (ইনসুলিন রক্তে গ্লুকোজের মাত্রা নিয়ন্ত্রণ করে)

৩ ইনসুলিন তখন সেই গ্লুকোজকে বিভিন্ন ধরণের কোষ যেমন-লিভার, মাংসপেশী, চর্বি কোষে পৌঁছে দেয় এবং রক্তের গ্লুকোজকে গ্রহণ করতে সাহায্য করে ।

৪ কোষে গ্লুকোজ প্রবেশের পরে তা শক্তি হিসাবে ব্যবহৃত হয় এবং এভাবে রক্তে গ্লুকোজের মাত্রা একটি নির্দিষ্ট সীমার মধ্যে থাকে।

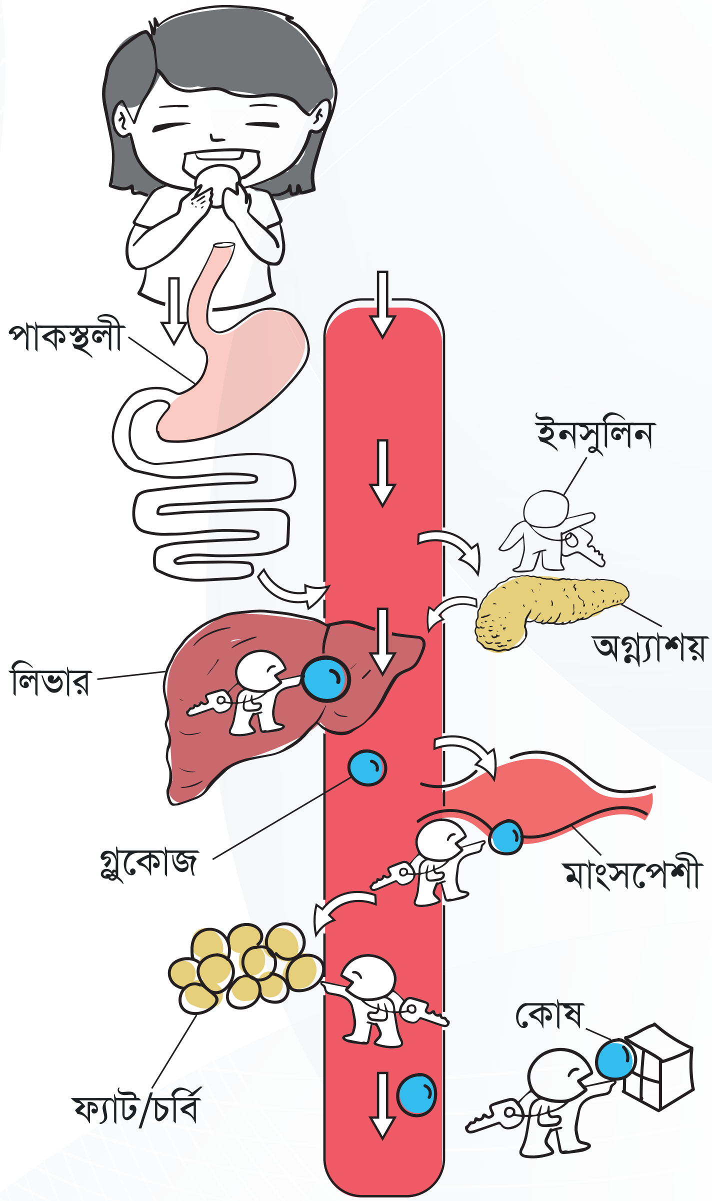

যাদের ডায়াবেটিস নেই তাদের ক্ষেত্রে এমনটি ঘটে থাকে।

‘‘ইনসুলিন যখন শরীরের বিভিন্ন কোষে গ্লুকোজকে বহন করে নিয়ে যায় তখনই তা শক্তির জন্য ব্যবহৃত হতে পারে। ইনসুলিনই একমাত্র হরমোন যা রক্তে গ্লুকোজের মাত্রা নিয়ন্ত্রণ করতে পারে।”

“যাদের ডায়াবেটিস আছে এবং রক্তে গ্লুকোজের মাত্রা ধারাবাহিকভাবে বাড়তে থাকে, তাদের ক্ষেত্রে যেমনটি ঘটে থাকে”

রক্তে যখন গ্লুকোজের মাত্রা ধারাবাহিক এবং নিয়ন্ত্রণহীনভাবে বাড়তে থাকে তখন রক্তের সেই বাড়তে থাকা গ্লুকোজকে নিয়ন্ত্রণ করার জন্য অগ্ন্যাশয় একনাগারে ইনসুলিন তৈরী করতে করতে ক্লান্ত হয়ে পড়ে।

অগ্ন্যাশয় ক্লান্ত হয়ে পড়ে কারণ এটি প্রচুর পরিমাণে ইনসুলিন তৈরি করার চেষ্টা করে।

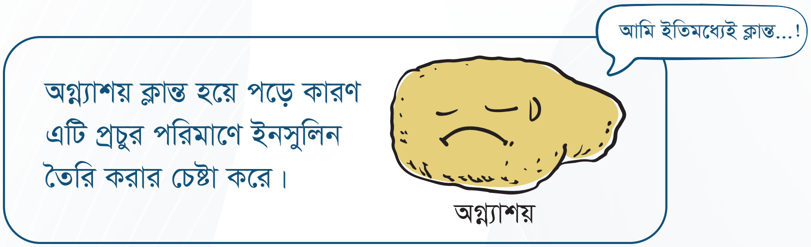

এভাবে, অগ্ন্যাশয় একনাগারে ইনসুলিন তৈরী করতে করতে খুব ক্লান্ত হয়ে পড়ার কারণে পর্যাপ্ত কর্মক্ষম ইনসুলিন তৈরী হওয়া কমে যায়।

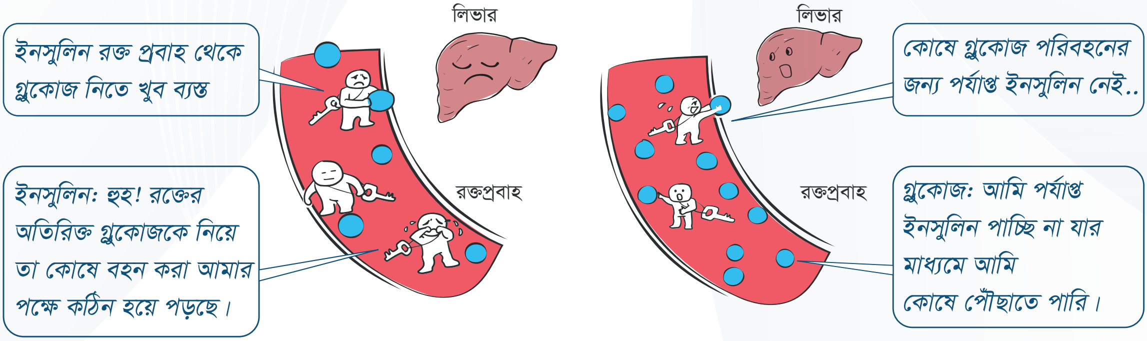

এই অবস্থায় পর্যাপ্ত কর্মক্ষম ইনসুলিন তৈরী না হওয়ার কারণে শরীরের বিভিন্ন কোষে সঠিকভাবে গ্লুকোজ পৌঁছানো কঠিন হয়ে পড়ে।

এই অবস্থায় রক্তে গ্লুকোজের মাত্রা বেড়ে যায়।

পরিণতি

রক্তে গ্লুকোজের মাত্রার ক্রমাগত বৃদ্ধি → হাইপারগ্লাইসেমিয়া → হাইপারগ্লাইসেমিয়া চলতে থাকে → ডায়াবেটিস তৈরী হয়।

দ্রষ্টব্য: ইনসুলিনের অভাবের কারণে আমিষ এবং চর্বি বিপাকও কঠিন হয়ে পড়ে।

ডায়াবেটিসের মৌলিক চিকিৎসাসমূহ

পর্যাপ্ত প্রোটিন গ্রহণ ← **ডায়েট** → পর্যাপ্ত ক্যালরি গ্রহণ

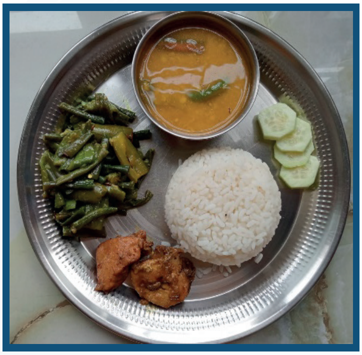

যে নিয়মে খাবার গ্রহণ করতে হবে..................

১. প্রথমে শাকসবজি (আঁশ), তারপর মাংস অথবা মাছ (প্রোটিন), এবং ভাত (কার্বোহাইড্রেট)।

২. ধীরে ধীরে এবং ভালোভাবে চিবিয়ে খেতে হবে।

৩. খাওয়ার সময় মনোযোগ সহকারে খেতে হবে।

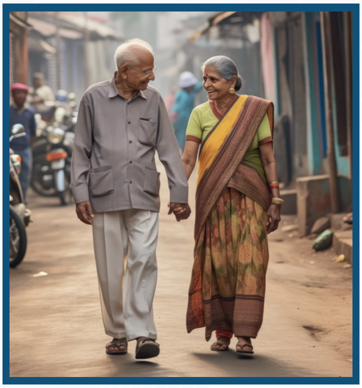

মাংসপেশী বর্ধক ← **ব্যায়াম থেরাপি** → অ্যারোবিক ব্যায়াম

আপনাকে যে নিয়মগুলো মেনে চলতে হবে...............

১. একটানা বসে কাজ না করে, কাজের ফাঁকে মাঝে মাঝে হাঁটতে হবে।

২. লিফটের পরিবর্তে সিঁড়ি ব্যবহার করার অভ্যাস তৈরী করতে হবে।

৩. খাওয়ার পর হালকাভাবে একটু হাঁটা-হাঁটি করতে হবে।

চিকিৎসা..................

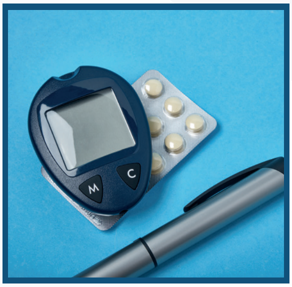

১. কিভাবে, কখন ওষুধ খেতে হবে, এবং সঠিকভাবে ওষুধ না খেলে তার কি প্রভাব হতে পারে তা জানা জরুরী।

২. হাইপোগ্লাইসেমিয়া/রক্তে গ্লুকজের মাত্রা কমে গেলে কি করণীয় তা জানতে হবে।

৩. অসুস্থ দিনগুলোতে কি কি করণীয় সেগুলো জানতে হবে।

ভুলে যাওয়া উচিৎ নয়.....

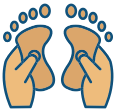

পায়ের যত্ন

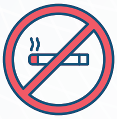

ধুমপান স্বাস্থ্যের জন্য ক্ষতিকর

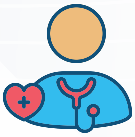

নিয়মিত ডাক্তারের পরামর্শ

সঠিক বি.এম.আই. (BMI) এবং দৈনন্দিন কতটুকু ক্যালরি/শক্তি প্রয়োজন সে সম্পর্কে জানুন

বি.এম.আই (BMI): উচ্চতা অনুযায়ী ওজন কেমন হওয়া উচিৎ এবং শরীরে কী পরিমাণ বাড়তি ফ্যাট/চর্বি রয়েছে ও সেই সাথে স্বাস্থ্যঝুঁকির প্রবণতা জানতে বি.এম.আই নির্ণয় করা হয়।

[যেভাবে বি.এম.আই. নির্ধারণ করতে হয়- বি. এম. আই.(BMI)=ওজন/উচ্চতা^{২} (কেজি/মি^{২})]

বি.এম.আই চার্ট

- কম ওজন <১৮.৫ কেজি/মি^{২}
- স্বাভাবিক ওজন ১৮.৫-২৩ কেজি/মি^{২}
- বেশি ওজন ২৩-২৭.৫ কেজি/মি^{২}
- মেদবহুল ≥২৭.৫ কেজি/মি^{২}

একজন ব্যক্তির উচ্চতা ১৬৬ সে.মি /৫'৫"
এবং তার ওজন ৫৫ কেজি।
বি.এম.আই = ওজন/উচ্চতা^{২} (কেজি/মি^{২})
= ৫৫/(১.৬৬)^{২}
=১৯.৯ বা ২০ (কেজি/মি^{২})

১৬৬/১০০=১.৬৬ মি.

সূত্রঃ *https://www.researchgate.net/figure/Classification-of-weight-according-to-BMI-for-Caucasian-South-Asian-and-Chinese_tbl1_340960106, April, 2020*

দৈনিক শক্তি (ক্যালরি) গ্রহণঃ আদর্শ শরীরের ওজন X ক্যালরি ফ্যাক্টর

একজন ব্যক্তির বি.এম.আই. (BMI) কত এবং তিনি কি ধরণের শারীরিক পরিশ্রম করেন তার উপরে নির্ভর করে ক্যালরি ফ্যাক্টর ২০ ক্যালরি/কেজি থেকে ৪৫ ক্যালরি/কেজি হিসেবে পরিবর্তিত হয়।

আদর্শ শরীরের ওজন = উচ্চতা (সে.মি.)-১০০

ক্যালরি ফ্যাক্টর হলো, একজন ব্যক্তির প্রতি কেজি ওজনের জন্য দৈনন্দিন কি পরিমাণ ক্যালরি প্রয়োজন তার হিসাব

সূত্রঃ ডায়াবেটিস মেলিটাসের জাতীয় গাইডলাইন/নির্দেশিকা; প্রথম সংস্করণ, আগস্ট, ২০২৩।

একজন ব্যক্তির ওজন ৫৫ কেজি। তিনি স্বাভাবিক পরীশ্রম করেন এবং তার বি.এম.আই. ২০ (কেজি/মি^{২})। এজন্য তার ক্যালরি ফ্যাক্টর আনুমানিক ৩০ ক্যালরি/কেজি/দিন।

দৈনিক শক্তি (ক্যালরি) গ্রহণঃ আদর্শ শরীরের ওজন ক্যালরি ফ্যাক্টর
=৫৫ কেজি X ৩০ ক্যালরি/কেজি/দিন
= ১৬৫০ ক্যালরি/দিন

ডায়েট/খাবার সম্পর্কে জানুন

সুষম খাদ্য চার্ট

আপনার হাতের তালুতে যে পরিমাণ ডিম, মাছ বা মাংসের টুকরোর জায়গা হয় সে পরিমাণ ডিম, মাছ বা মাংস প্রতি খাবারে খেতে পারবেন ।

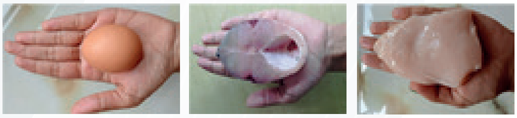

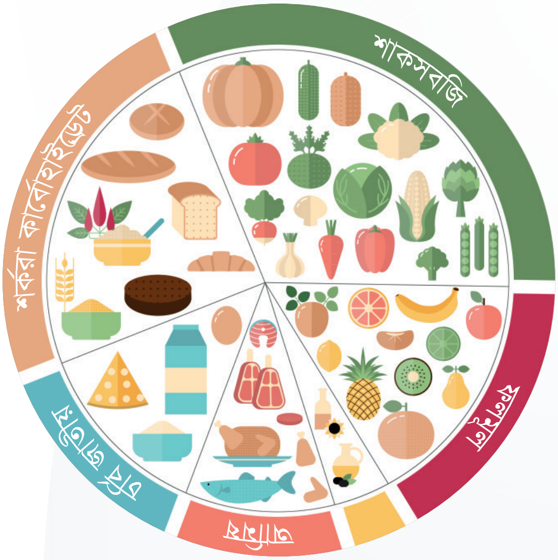

দৈনিক

কার্বোহাইড্রেট/শর্করা	- ৫০%
প্রোটিন/আমিষ	- ১০-২০%
ফ্যাট/চর্বি	-৩০%

প্লেটে প্রস্তুতকৃত খাবারে প্রধানত সবজি, ভাত বা রুটি এবং মাংস বা মাছ বা ডিম থাকতে হবে।

সব ধরনের তেলে একই ক্যালরি থাকে।
সব ধরনের তেল/ঘি = ১ চা চামচ = ৪৫ ক্যালরি।

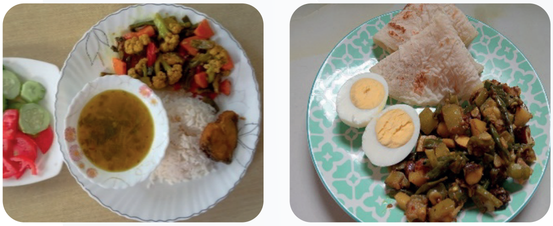

সুষমভাবে বিভিন্ন ধরনের খাবার খান

(স্টেজ-৩ ডায়াবেটিক নেফ্রোপ্যাথি থাকলে আমিষ খাওয়া সীমাবদ্ধ করতে হবে)

এই অবস্থায় নেফ্রোলজিস্টের সাথে যোগাযোগ করতে হবে এবং একজন রেজিস্টার্ড ডায়েটিশিয়ানের পরামর্শ নিতে হবে যিনি কিডনিতে বিশেষজ্ঞ।

প্রতিদিন খাবারের সাথে ৫ গ্রাম বা তার কম লবণ খেতে হবে। (পরিমাপটি ডায়াবেটিক নেফ্রোপ্যাথির স্টেজ অনুসারে পরিবর্তনশীল)

পরিমাপের জন্য আপনি নিম্নলিখিত পাত্রগুলি ব্যবহার করতে পারেন

১ কাপ = ১৫০ মিলি = ১২০ গ্রাম
১ চা চামচ = ৫ গ্রাম
১ টেবিল চামচ = ১৫ গ্রাম

সূত্র: ডায়াবেটিক গাইড বই, বাংলাদেশ ডায়াবেটিক সোসাইটি।

দৈনন্দিন খাবারের নির্দেশনা

প্রতিদিন খাবারে আনুমানিক **১৪০০ ক্যালরি** বজায় রাখতে নিম্নলিখিত ডায়েট অনুসরণ করতে পারেন।

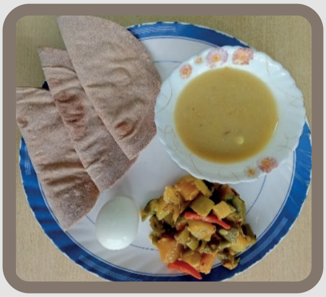

সকালের নাস্তা

২ টি আটার রুটি= ১৫০ ক্যালরি
১ টি সিদ্ধ ডিম= ৭৫ ক্যালরি
রান্নাকরা সবজি ইচ্ছেমতো

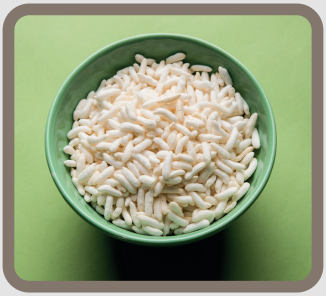

সকাল ১১ টা বা মধ্য-মর্নিং

২ কাপ মুড়ি= ১৫০ ক্যালরি

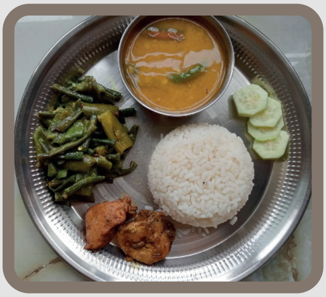

দুপুরের খাবার

২ কাপ ভাত= ৩০০ ক্যালরি
২ টুকরা মাংস= ৬০ গ্রাম বা আনুমানিক ১০০ ক্যালরি
১ কাপ মাঝারী ঘন/
২০ গ্রাম ডাল=২৪ ক্যালরি
রান্নাকরা সবজি ইচ্ছেমত

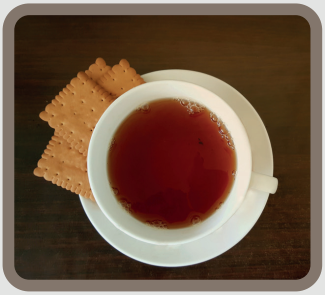

৫-৬ টা বা সন্ধ্যার নাস্তা

৪ টি লেক্সাস বিস্কুট= ৭৫ ক্যালরি
১ কাপ চিনি ছাড়া চা

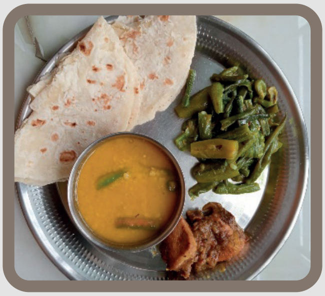

রাতের খাবার

২ টি রুটি= ১৫০ ক্যালরি
১ কাপ মাঝারী ঘন/
২০ গ্রাম ডাল= ২৪ ক্যালরি
২ টুকরা মাংস= ৬০ গ্রাম বা আনুমানিক ১০০ ক্যালরি
রান্নাকরা সবজি ইচ্ছেমত

- রাতের খাবার ৮-৯ টার মধ্যে খেতে হবে।
- রাতে খাওয়ার কমপক্ষে দেড় থেকে দুই ঘন্টা পড়ে ১ কাপ দুধ খাওয়া যাবে।

রান্নার তেল ২০ মিলিলিটার

খাওয়ার যে নিয়ম অনুসরণ করলে রক্তে গ্লুকোজের মাত্রা দ্রুত বাড়বে না । (৩টি উপায়)

১. প্রথমে শাকসবজি, তারপর প্রোটিন/আমিষ এবং শেষে কার্বোহাইড্রেট/শর্করা যেমন ভাত বা রুটি খেতে হবে।

ভাত বা রুটিতে প্রচুর পরিমাণে শর্করা পাওয়া যায় যা খাওয়ার পরে রক্তে খুব তাড়াতাড়ি গ্লুকোজের মাত্রা বাড়ায়। শর্করার মধ্যে রয়েছে চিনি এবং ফাইবার। কাঁচা শাকসবজি যেমন সালাদ, এবং রান্না করা শাকসবজি উভয়ই খেতে হবে।

২. ধীরে ধীরে খাবার খেতে হবে

তাড়াহুড়ো করে খাওয়ার ফলে রক্তে গ্লুকোজের মাত্রা দ্রুত বৃদ্ধি পায়। তাড়াহুড়ো করে খেলে অপরিপূর্ণ অনুভূতি হয় এবং অতিরিক্ত খাওয়ার ইচ্ছা হতে পারে। এজন্য ধীরে ধীরে উপভোগ করে খাবার খেতে হবে।

৩. খাবার ভাবেভাবে চিবিয়ে খেতে হবে

খাবার ভালোভাবে চিবিয়ে খেলে রক্তে গ্লুকোজের মাত্রা ধীরে ধীরে বাড়ে। এতে মস্তিষ্কে রক্ত প্রবাহও উন্নত হয়।

শুধু শর্করা জাতীয় খাবারই নয়, কিন্তু খাবারের অন্যান্য উপাদানও রক্তে গ্লুকোজের মাত্রা বাড়ায়।

শর্করা রক্তে গ্লুকোজের মাত্রা ১০০% বাড়ায়।

আমিষ খাওয়ার কিছু সময় পরে রক্তে গ্লুকোজের মাত্রা ৫০% বাড়ায়।

এমনকি চর্বি জাতীয় খাবার খাওয়ার পরেও, সময়ের সাথে সাথে রক্তে গ্লুকোজের মাত্রা ধীরে ধীরে বাড়ে।

সুত্রঃ টেলিনার্সিং ডায়াবেটিক গাইডলাইন বই, জাপান।

উদাহরণঃ বিশেষ করে মিষ্টি বা চিনি জাতীয় কিছু না খেয়ে থাকলেও সকালে খাওয়ার আগে ব্লাড সুগার পরীক্ষা করে দেখা গেলো রক্তে গ্লুকোজের মাত্রা বেশি। তাহলে বুঝতে হবে ডায়াবেটিক ব্যাক্তিটি আগের রাতে ঠিক ঘুমানোর আগে চর্বি জাতীয় কোনো খাবার খেয়েছেন।

যে খাবারগুলো এড়িয়ে চলতে হবে

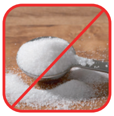
চিনি

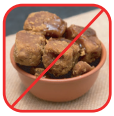
গুড়

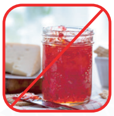
জেলি

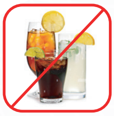
সফট্ ড্রিংক

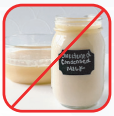
কনডেন্স মিল্ক

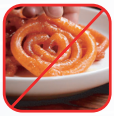
জিলাপী

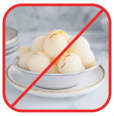
মিষ্টি

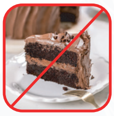
কেক

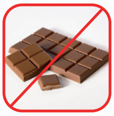
চকলেট

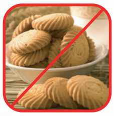
মিষ্টি বিস্কুট

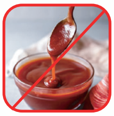
সস

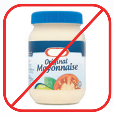
মিয়োনিজ

নিম্নলিখিত যেকোনো একটি ফল বা ফলের অংশটুকু দিনে ১টা করে খাওয়া যাবে

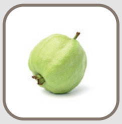
পাঁকা পেয়ারা

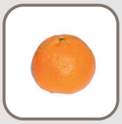
কমলা

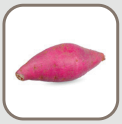
কেশর আলু

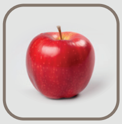
আপেল

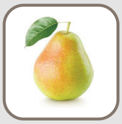
নাশপাতি

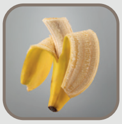
অর্ধেক কলা

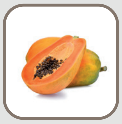
পেঁপে

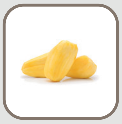
কাঁঠাল

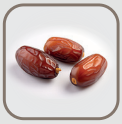
৩টি খেজুর

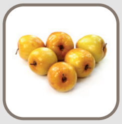
মিষ্টি কুল

স্বাস্থ্য ভালো রাখার জন্য প্রতিদিন কিছু টক জাতীয় ফল খেতে হবে।

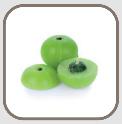
আমলকি

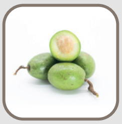
জলপাই

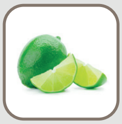
লেবু

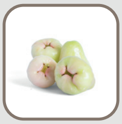
জামরুল

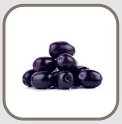
কালজাম

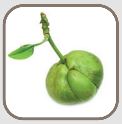
চালতা

রমজান মাসে যে-সকল নিয়ম মেনে চলতে হবে

রমজানের কমপক্ষে ২ মাস আগে ডাক্তারের সাথে পরামর্শ করতে হবে ।

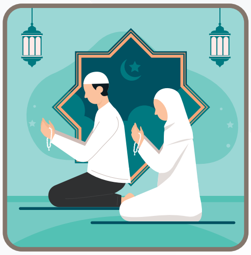

ঔষধ খাওয়ার সময়সূচি

সকালের ঔষধের ডোজ : ইফতারের সময়
রাতের ঔষধের ডোজ (অর্ধেক) : সেহেরির সময়

রমজান মাসে কি খেতে হবে আর কিভাবে খেতে হবে?

ইফতার : শরবত; (ইসবগুল, তোকমা, তেতুল, লেবু, কাঁচা আমের রস), চিনি ছাড়া সতেজ ফলের রস। পূর্বউল্লিখিত সকালের নাস্তার সমপরিমাণ খাবার খেতে হবে।

রাতের খাবার: পূর্বউল্লিখিত রাতের খাবারের সমান খাবার খেতে হবে।

সেহেরি : সেহেরির শেষ সময়ে খাবার খাওয়া ভালো। পূর্বউল্লিখিত দুপুরের খাবারের সমান খাবার খেতে হবে। খাবারের তালিকায় ভাত এবং ফল অথবা ভাত এবং দুধ একসাথে নেওয়া যাবে তবে একই সাথে খাবারের তালিকায় ভাত, ফল এবং দুধ নেওয়া যাবে না।

রোজা ভেঙে ফেলতে হবে; যদি রক্তে গ্লুকোজের মাত্রা ৩.৯-এর নিচে বা ১৬.৭-এর বেশি হয়।

সূত্র: ডায়াবেটিক গাইড বই, বাংলাদেশ ডায়াবেটিক সোসাইটি।

দ্রষ্টব্য: রমজান মাসে শারীরিক ব্যায়াম করার দরকার নেই। শুধু দৈনন্দিন কাজগুলো সঠিকভাবে করলেই হবে।

ব্যায়াম সম্পর্কে জানুন

যাদের কিডনি, হার্ট এবং স্নায়ুতন্ত্রের কোনো জটিলতা নেই সেই সাথে যাদের ব্লাড প্রেসার/রক্তচাপের মাত্রা স্বাভাবিক তারা সব ধরনের কাজ করতে পারবে।

ব্যায়ামের কার্যকারিতা বাড়ানোর উপায়ঃ

- প্রতিদিন ৩০ মিনিট করে সপ্তাহে কমপক্ষে ৫ দিন ব্যায়াম করা শরীরের জন্য উপকারী।
- খাবারের ১ থেকে দেড় ঘন্টা পরে রক্তে গ্লুকোজের মাত্রা সর্বোচ্চে পৌঁছায়।
- খাওয়ার পর ব্যায়াম করলে রক্তে গ্লুকোজের মাত্রা হঠাৎ করে কমে যেতে পারে।
- এজন্য প্রধান খাবার যেমন সকাল, দুপুর এবং রাতের খাবারের অন্তত ১ ঘন্টা পর ব্যায়াম করা উচিৎ।

প্রতিদিনের ব্যায়ামের জন্য একটি নিদিষ্ট সময় নির্ধারণ করতে হবে। প্রতিদিন একই ধরণের ব্যায়াম করতে হবে।

অনেকের ক্ষেত্রে খাওয়ার পরপরই রক্তে গ্লুকোজের মাত্রা হঠাৎ বেড়ে যায় যাকে পোস্টপ্রান্ডিয়াল হাইপারগ্লাইসেমিয়া বা ব্লাড সুগার স্পাইক বলে আবার খুব তাড়াতাড়ি কমেও যায়। তবে সাধারণ অবস্থায় রক্তে গ্লুকোজের মাত্রা ধীরে ধীরে বাড়ে এবং ধীরে ধীরে কমে।

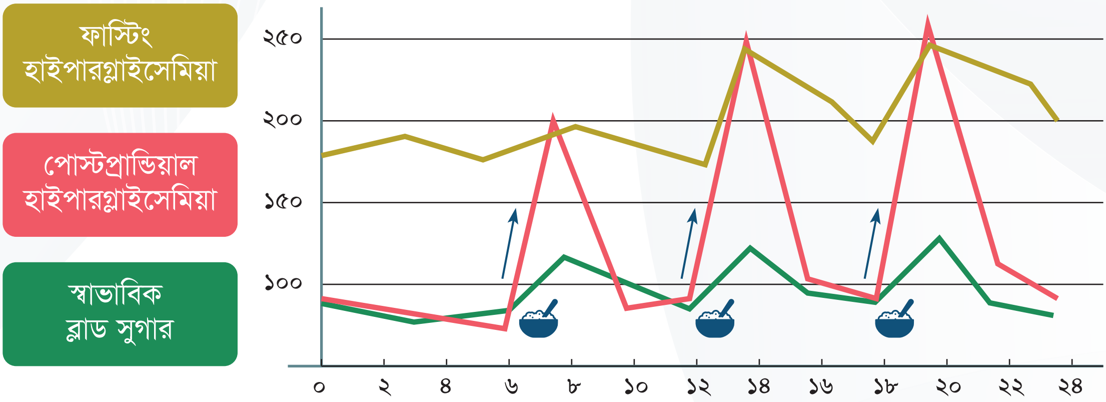

এর কারণ হলো, শরীরের রক্তে গ্লুকোজের মাত্রা কমানোর চেষ্টায় অগ্ন্যাশয় প্রচুর পরিমাণে ইনসুলিন নিঃসরণ করলেও গ্লুকোজ সঠিকভাবে শরীরের কোষে প্রবেশ করে না।

“ব্লাড সুগার স্পাইক”- এ অবস্থা অনিয়ন্ত্রিতভাবে চলতে থাকলে অ্যাথেরোস্ক্লেরোসিস বা রক্তনালীর সংকোচন হতে পারে যা থেকে পরবর্তীতে হার্ট-অ্যাটাক এবং ক্যানসারের ঝুঁকি রয়েছে।

যদি খাওয়ার পরপরই রক্তে গ্লুকোজের মাত্রা হঠাৎ বেড়ে যাওয়ার পূর্ব অভিজ্ঞতা থাকে, তবে খাওয়ার পর কিছু সময় হালকা হাঁটাহাঁটি করা সহায়ক এবং খাওয়ার কমপক্ষে ২-৩ ঘন্টা পরে ঘুমাতে যেতে হবে।

নিয়মিত শারীরিক ব্যায়ামের লক্ষ্যঃ

প্রতি সপ্তাহে ২ ঘন্টা ৩০ মিনিট শারীরিক ব্যায়াম করতে হবে। যেমনঃ সপ্তাহে ৫ দিন প্রায় ৩০ মিনিট দ্রুত হাঁটা।

সপ্তাহে ৫ দিন ব্যায়াম করা সম্ভব না হলে অন্তত সপ্তাহে ৩ দিন ব্যায়াম করা প্রয়োজন। (ব্যায়ামের পরে ২ দিন পর্যন্ত তার প্রভাব থেকে যায়।)

দ্রষ্টব্যঃ খালি পায়ে ব্যায়াম করা উচিৎ না এবং ব্যায়ামের সময় পর্যাপ্ত পানি খেতে হবে।

যদি ব্যায়ামের পূর্ব কোনো অভিজ্ঞতা না থাকে তাহলে ধীরে ধীরে সেই অভ্যাস গড়ে তুলতে হবে।

ব্যায়ামের ধাপসমূহঃ

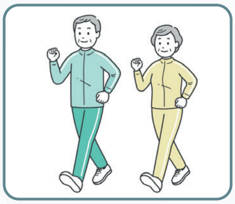

১. ওয়ার্মিং আপ বা আস্তে আস্তে হাঁটাঃ (৫-১০ মিনিট)

দুর্ঘটনা এড়াতে প্রথমে আস্তে আস্তে হেঁটে ব্যায়াম শুরু করতে হবে।
যেমনঃ হালকা হেঁটে ব্যায়াম শুরু করলে তা শরীরকে সতেজ করবে এবং পরবর্তী ব্যায়ামের জন্য প্রস্তুত করবে।

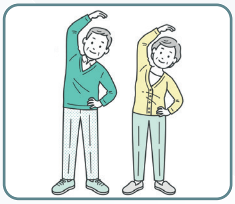

২. স্ট্রেচিং বা হালকা শরীরের ব্যায়ামঃ (৫-১০ মিনিট)

প্রথমেই দ্রুত না করে ধীরে ধীরে মাংসপেশী প্রসারিত করতে হবে।
উদাহরণঃ পিঠের ব্যথা প্রতিরোধের জন্য স্ট্রেচিং, ইত্যাদি।

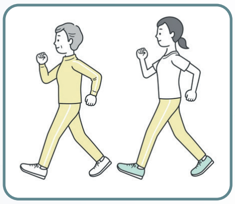

৩. শক্তিসহ মাংসপেশীর ব্যায়ামঃ (৩০ মিনিট)

যতক্ষণ না সামান্য ঘাম হয় ততক্ষণ এই ব্যায়ামটি করতে হবে।
উদাহরণঃ চেয়ারে উঠা-বসা, দ্রুত হাঁটা, সিঁড়ি বেয়ে উঠা-নামা, ইত্যাদি।

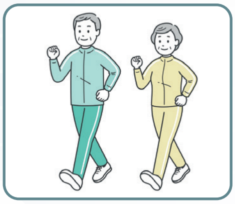

৪. কুল-ডাউন বা আস্তে আস্তে হাঁটাঃ (৫-১০ মিনিট)

ব্যায়ামের পরে দুর্ঘটনা এবং পেশী ব্যথা প্রতিরোধ করার জন্য এটি করা হয়। ব্যায়ামের সময় বেড়ে যাওয়া নিঃশ্বাস শান্ত না হওয়া পর্যন্ত হালকা ভাবে ব্যায়াম করতে হবে।

যারা দীর্ঘসময় বসে কাজ করেন তাদের ক্ষেত্রে একটানা বসে না থেকে ৩০ মিনিট পরপর হালকাভাবে হাঁটতে হবে।

নিম্নলিখিত অবস্থাতে ব্যায়াম ঝুঁকিপূর্ণ তাই এসকল অবস্থাতে ব্যায়াম এড়িয়ে চলতে হবেঃ

- রক্তে গ্লুকোজের মাত্রা স্বাভাবিকের চেয়ে বেশি, >১৬.৭ মিলিমোল/লি।
- রক্তচাপ স্বাভাবিকের চেয়ে বেশি, ১৬০-১০০ মিলিমিটার মার্কারী (mmHg)।
- হৃদস্পন্দন বা হার্ট-রেট স্বাভাবিকের (৬০-১০০ বিট/মিনিট) চেয়ে দ্রুত হয়।
- মাথা ঘুরছে এমন মনে হলে/ঘুমের অভাব।
- অস্বাভাবিকভাবে ক্লান্ত বোধ করা।
- হাড়ের জয়েন্ট এবং পেশীতে তীব্র ব্যথা অনুভব করা।
- হঠাৎ কোনো সমস্যা দেখা দিলে যেমন-মাথাব্যথা, পেটে ব্যথা, ডায়রিয়া এবং জ্বর, ঠান্ডা-কাশি, হার্ট-অ্যাটাক, ইত্যাদি।

বুকে ব্যথা অনুভব হলে ব্যায়াম বন্ধ করে বিশ্রাম নিতে হবে বা শুয়ে থাকতে হবে। কিছুক্ষণ বিশ্রামের পরেও যদি ব্যথা না কমে, তাহলে দেরি না করে হাসপাতালে যেতে হবে।

ব্যথার ধরণঃ ব্যথা শুধুমাত্র বুকের এলাকায় নয়, বুক থেকে (উভয়) কাঁধ এবং হাত পর্যন্ত বা বুক থেকে কোমড় এবং পিঠ পর্যন্ত বিস্তৃত এলাকা জুড়েও দেখা দিতে পারে।

হৃদপিন্ডে ব্যথা, হার্ট-অ্যাটাক, ডায়াবেটিক নেফ্রোপ্যাথিজনিত কোনো সমস্যা থাকলে ডাক্তারের সাথে ব্যায়ামের নিয়মাবলী বিষয়ে পরামর্শ করতে হবে।

দ্রষ্টব্য ১ : পিঠে এবং হাঁটুতে নিউরোপ্যাথি বা অস্টিওআথ্রাইটিস থাকলে হাঁটা, জগিং বা ট্রেডমিল করা যাবে না। এক্ষেত্রে সাঁতার কাটা এবং সাইকেল চালানো উপকারী। ডায়াবেটিক রেটিনোপ্যাথি বা চোখের সমস্যা থাকলে সাঁতার, হাঁটা বা সাইকেল চালানো উপকারী ব্যায়াম। ভারী ওজন তোলা যাবে না।

বিশেষ অবস্থাঃ ডায়াবেটিক রেটিনোপ্যাথি, পায়ে ক্ষত, গর্ভাবস্থা।

দ্রষ্টব্য ২ : যারা খুব পরিশ্রমী (যেমন-খেলোয়ার এবং শ্রমিক) তাদের বাড়তি ব্যায়াম না করলেও চলবে।

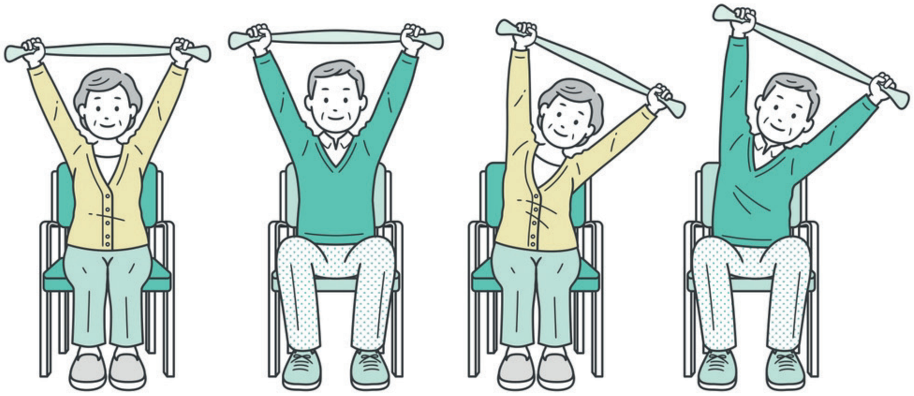

পায়ের যত্ন

সাধারণত খুব গুরুত্ব সহকারে নিয়মিত পায়ের যত্ন নেওয়া সম্ভব হয়ে ওঠে না। ডায়াবেটিসে আক্রান্ত ব্যক্তিরা সঠিকভাবে পায়ের যত্ন না নিলে ডায়াবেটিক নিউরোপ্যাথি এবং পায়ে ছড়িয়ে থাকা রক্তনালীতে সমস্যা হয়ে থাকে যাকে পেরিফেরাল ভাসকুলার ডিজিস বলা হয়। তাই সু-স্বাস্থ্য নিশ্চিত করার জন্য পায়ের যত্ন গুরুত্বপূর্ণ।

ডায়াবেটিক নিউরোপ্যাথির উচ্চ ঝুঁকি থাকে, যদি-

- পায়ের পাতায় অনুভূতি কমে যায়
- অস্বাভাবিক কোনো অনুভূতি হয়
- জ্বালাপোড়া, চিনচিন করে ওঠা, পিন বা সূঁচ ফোঁটার মতো কোনো অনুভূতি হয়

প্রতিদিন অন্তত একবার পায়ের যত্ন নিতে হবে

নিয়মিত পায়ের যত্নঃ

১. হালকা গরম পানি দিয়ে পা ধুয়ে নিতে হবে

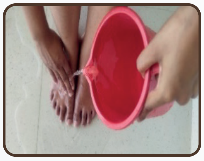

পায়ে গরম পানি ঢালার আগে প্রথমে হাত দিয়ে পানির তাপমাত্রা পরীক্ষা করে দেখতে হবে যেনো এটি খুব গরম না হয়। পায়ের আঙুলের মধ্যে ভালো করে ধুয়ে নিতে হবে।

২. একটি শুকনো এবং নরম তোয়ালে দিয়ে পা মুছতে হবে

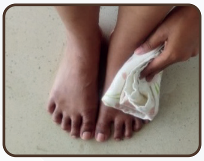

পায়ের আঙুলের মধ্যে ভালোভাবে মুছতে হবে।

৩. লোশন লাগানো

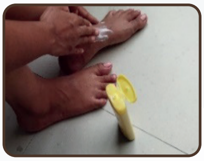

এমন লোশন বেছে নিতে হবে যা আঠালো নয়।
পায়ের আঙুলের মধ্যে লোশন লাগানো যাবে না।

৪. পায়ের ত্বকের অবস্থা সাবধানে পর্যবেক্ষণ করতে হবে

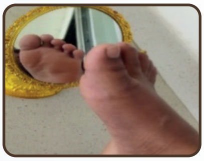

• পায়ের নখ কি সাদা এবং পুরু? • জুতার ফোস্কা, • শুকনাভাব • শক্তভাব
• ত্বকের রঙ ও গঠন • ফোলাভাব • ফাটল • পায়ের নখ এবং নখের রঙ

৫. জুতার সাথে সবসময় পরিষ্কার মোজা পড়তে হবে

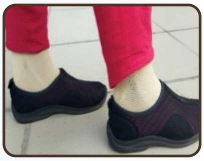

মোজা পড়ার আগে দেখে নিতে হবে যেনো তার ভিতরে ধারালো কিছু না থাকে। মোজা যেনো ভেজা না থাকে সেদিকেও খেয়াল রাখতে হবে।

৬. খালি পায়ে হাঁটা যাবে না

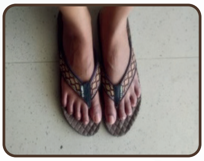

যদি জুতা ব্যবহার করা সম্ভব না হয় তাহলে অন্তত পায়ে যেকোনো ক্ষত এড়াতে হাঁটার সময় সবসময় স্যান্ডেল ব্যবহার করতে হবে।

HbA1c এবং হাইপোগ্লাইসেমিয়া সম্পর্কে জানুন

HbA1c (গ্লাইকেটেড হিমোগ্লোবিন)

রক্তে HbA1c (%)-এর মাত্রা যত বেশি হবে, রক্তে গ্লুকোজের মাত্রা নিয়ন্ত্রণে তত বেশি জটিলতা তৈরী হবে।

HbA1c হলো রক্তের লোহিত কণিকার হিমোগ্লোবিনের সাথে লেগে থাকা গ্লুকোজ। লোহিত রক্তকণিকা রক্তনালীর মধ্যে প্রবাহিত হয় এবং হিমোগ্লোবিনের সাথে লেগে থাকা গ্লুকোজও এভাবে রক্তের মধ্যে চলতে থাকে। তাই, রক্তে গ্লুকোজ যত বেশি, HbA1c মান তত বেশি।

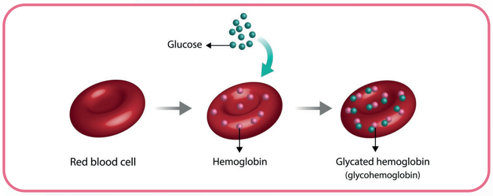

HbA1c, বিগত ৩ মাসের রক্তে গ্লুকোজের গড় মাত্রা প্রকাশ করে।

বিভিন্ন অবস্থা অনুযায়ী রক্তে HbA1c (%)-এর মাত্রা যেমন থাকতে হবে...

গর্ভধারণের পূর্বে	৬.৫%-এর নিচে
প্রাপ্তবয়স্ক ব্যক্তি (গর্ভবতী নয়)	৭%-এর নিচে
বয়স্ক ব্যক্তি যাদের অন্য আরও অসুস্থতা আছে	৭-৮%

রক্তে গ্লুকোজের স্বাভাবিক লক্ষ্যমাত্রা

- সকালে ঘুম থেকে উঠে খালি পেটে রক্তে গ্লুকোজের স্বাভাবিক মাত্রা — ৭.০ মিলিমোল/লি এর নিচে
- খাবারের ১-২ ঘন্টা পরে রক্তে গ্লুকোজের স্বাভাবিক মাত্রা — ১০ মিলিমোল/লি এর নিচে

ডায়াবেটিসের জটিলতা প্রতিরোধ করতে রক্তে গ্লুকোজের মাত্রা সহ নিম্নোক্ত বিষয়গুলো নিয়ন্ত্রণ করতে হবে ।

ট্রাইগ্লিসারাইড (Triglycerides)	১৫০ মিলিগ্রাম/ডেল এর নিচে
এইচডিএল (HDL)	পুরুষ: ৪০ মিলিগ্রাম/ডেল এর উপরে<br>মহিলা: ৫০ মিলিগ্রাম/ডেল এর উপরে
এলডিএল (LDL)	১০০ মিলিগ্রাম/ডেল এর নিচে, হার্টের সমস্যা থাকলে ৭০ মিলিগ্রাম/ডেল এর নিচে
কোলেস্টেরল (Cholesterol)	২০০ মিলিগ্রাম/ডেল এর নিচে
বিএমআই (BMI)	< ২৩ কেজি/মি^{২}
রক্তচাপ (Blood Pressure)	১৩০/৮০ মিমি মার্কারীর নিচে

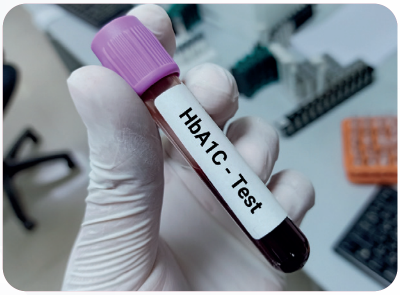

যাদের রক্তে গ্লুকোজের মাত্রা নিয়ন্ত্রণে আছে তাদেরকে অন্তত বছরে ২ বার HbA1c (%) পরীক্ষা করতে হবে।

যে ব্যক্তির এখনও জটিলতা নেই তার ক্ষেত্রে প্রযোজ্য।

সূত্র: ডায়াবেটিস মেলাইটাসের জাতীয় গাইডলাইন/নির্দেশিকা; প্রথম সংস্করণ, আগস্ট, ২০২৩।

একটি উদাহরণ: ২ জন ডায়াবেটিসে আক্রান্ত ব্যক্তির HbA1c ৭%। তাদের মধ্যে একজনের সারাদিনে রক্তে গ্লুকোজের মাত্রা মোটামুটি স্থির থাকে এবং অন্যজনের সারাদিনে রক্তে গ্লুকোজের মাত্রা অনেক বেশি ওঠানামা করে। এক্ষেত্রে যার সারাদিনে রক্তে গ্লুকোজের মাত্রা অনেক বেশি ওঠানামা করে তার বড় রক্তনালীতে ব্লক হওয়ার ঝুঁকি অন্য ব্যক্তির থেকে বেশি।

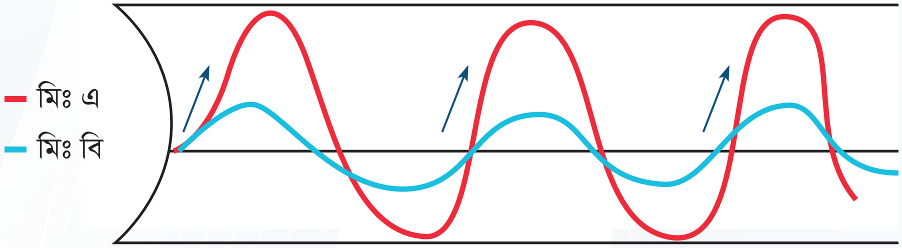

গ্রাফটি থেকে বোঝা যায় মিঃ বি-এর তুলনায় মিঃ এ রক্তনালীতে ব্লক হওয়ার ঝুঁকিতে রয়েছে।

দ্রষ্টব্য ১. লক্ষ্যটি শুধুমাত্র উপযুক্ত ডায়েট এবং ব্যায়াম থেরাপির মাধ্যমে অর্জন করা যেতে পারে, এমনকি কোনো পার্শ্বপ্রতিক্রিয়া যেমন হাইপোগ্লাইসেমিয়া হবে না এমনভাবে ড্রাগ থেরাপির মাধ্যমেও লক্ষ্যটি অর্জিত হতে পারে।

দ্রষ্টব্য ২. জটিলতা প্রতিরোধের উদ্দ্যেশ্যে, HbA1c-এর মান ৭% এর নিচে রাখতে নিয়ন্ত্রণের লক্ষ্য নির্ধারণ করতে হবে। অনুরূপ রক্তে গ্লুকোজের একটি স্বাভাবিক মাত্রার নির্দেশিকা হলো খালি পেটে রক্তে গ্লুকোজের মাত্রা ৭ মিলিমোল/লি. এর নিচে এবং খাবারের ২-ঘন্টা পরে রক্তে গ্লুকোজের মাত্রা ১০ মিলিমোল/লি. এর নিচে থাকবে।

দ্রষ্টব্য ৩. হাইপোগ্লাইসেমিয়া বা অন্যান্য পার্শ্বপ্রতিক্রিয়ার কারণে চিকিৎসা কঠিন হয়ে পড়লে একটি দৃঢ় লক্ষ্য ঠিক করতে হবে।

দ্রষ্টব্য ৪. সমস্ত লক্ষ্য মান প্রাপ্তবয়স্কদের জন্য, এবং গর্ভবতীরা এর বহির্ভূত।

সূত্র: জাপান ডায়াবেটিস সোসাইটি ডায়াবেটিস চিকিৎসা নির্দেশিকা ২০১৬-২০১৭।

হাইপোগ্লাইসেমিয়ার লক্ষণগুলো সনাক্তকরণের পাশাপাশি প্রয়োজনীয় ব্যবস্থাপনা

হাইপোগ্লাইসেমিয়ার অবস্থাটি বার বার ঘটতে থাকলে কিছু আচরণগত পরিবর্তন হয়, সেই সাথে বুদ্ধি প্রতিবন্ধকতা তৈরী হতে পারে এবং পরিণতিস্বরূপ হার্টের সমস্যা হতে পারে।

হাইপোগ্লাইসেমিয়া ঘটে যখন রক্তে গ্লুকোজের মাত্রা ৩.৯ মিলিমোল/লি-এর নিচে নেমে যায়

হাইপোগ্লাইসেমিয়ার লক্ষণ

ঝাপসা দৃষ্টি, অতিরিক্ত ক্ষুধা, দুর্বলতা, ক্লান্তি, শীতল ঘাম, বুক ধড়ফড় করা, কাঁপুনি, ফ্যাকাশে এবং লাল মুখ, অস্পষ্ট কথা।

হাইপোগ্লাইসেমিয়ার ধাপ সমূহ	
**১ম ধাপ :** ৩.৯ থেকে ৩ মিলিমোল/লি.-এর নিচে	এ অবস্থাতে নিজেই ব্যাবস্থা নেওয়া যায়
**২য় ধাপ :** ৩ মিলিমোল/লি.-এর নিচে	এ অবস্থাতে নিজেই ব্যাবস্থা নেওয়া যায়
**৩য় ধাপ :** এটি একটি ঝুঁকিপূর্ণ মুহূর্ত যে অবস্থায় জ্ঞান হারানো, অস্বাভাবিক আচরণ, খিঁচুনি, কোমা হতে পারে	

সূত্র: ডায়াবেটিস মেলাইটাসের জাতীয় গাইডলাইন/নির্দেশিকা; প্রথম সংস্করণ, আগস্ট, ২০২৩।

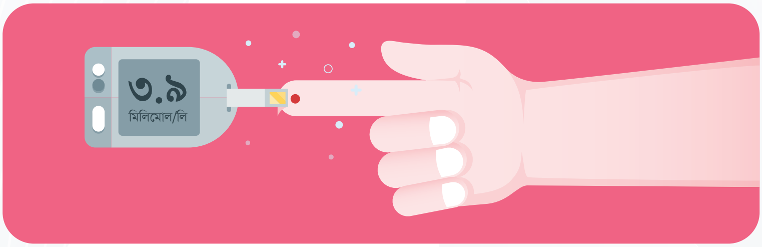

যেভাবে হাইপোগ্লাইসেমিয়া মোকাবেলা করতে হবে

যদি রোগী সচেতন থাকেন তখন যা করতে হবে;

- ১৫ গ্রাম কার্বোহাইড্রেট/শর্করা মানে ৩ চামচ চিনি/মধু খাওয়া যাবে।
- তারপর ১৫ মিনিট অপেক্ষা করে রক্তের গ্লুকোজ পরীক্ষা করতে হবে।
- রক্তের গ্লুকোজ স্বাভাবিক না হলে পুনরায় ১৫ গ্রাম শর্করা খেতে হবে। মনে রাখতে হবে যেন গ্লুকোজের মাত্রা ৫.৫ মিলিমোল/লি.-এর কম না হয়।

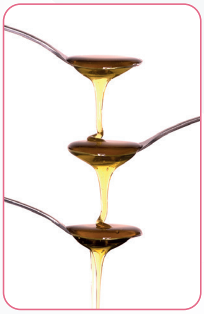

মনে রাখতে হবে; যারা নিয়মিত ডায়াবেটিসের ওষুধ (ট্যাবলেট) খেয়ে থাকেন তাদের শরীরে যেনো গ্লুকোজের ঘাটতি না হয়।

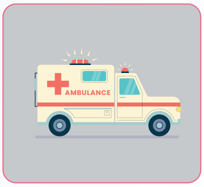

যদি রোগী জ্ঞান হারিয়ে ফেলেন তখন যা করতে হবে

আশেপাশের মানুষের সাহায্য চাইতে হবে। অচেতন ব্যক্তির মাড়িতে, ঠোঁটের ভিতরে এবং জিহ্বায় চিনি ঘষতে হবে।

যত দ্রুত সম্ভব একটি অ্যাম্বুলেন্স কল করতে হবে এবং হাসপাতালে নিয়ে যাওয়ার ব্যবস্থা করতে হবে। এ অবস্থায় মুখের মধ্যে কোনো পানীয় দেওয়া যাবে না, এতে শ্বাসনালীতে তা প্রবেশ করে শ্বাসরোধ হতে পারে।

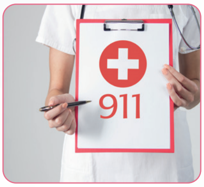

অসুস্থতার দিনগুলোতে করণীয়

অসুস্থতার দিনগুলোকে যেভাবে মোকাবেলা করতে হবে (যেমন জ্বর, বমি এবং ডায়রিয়া)

অসুস্থ দিনে রক্তে গ্লুকোজের মাত্রা নিয়ন্ত্রণে রাখা খুব কঠিন। ঠাণ্ডা, জ্বর, বমি এবং ডায়রিয়ার মতো সমস্যাগুলো ঝুঁকিপূর্ণ হতে পারে।

অসুস্থতার দিনগুলোতে মনে রাখতে হবে

- জ্বর (তাপমাত্রা ১০০° ফারেনহাইট বা ৩৮° সেলসিয়াস-এর বেশি), বমি, এবং ডায়রিয়ার কারণে প্রায়ই হাইপারগ্লাইসেমিয়া এবং কখনও কখনও হাইপোগ্লাইসেমিয়া হয়ে থাকে।
- অসুস্থতার আরেকটি লক্ষণ হলো একেবারেই খেতে না পারা অথবা পর্যাপ্ত খেতে না পারা।

যদি এমন একটি লক্ষণও থাকে, তাহলে ডাক্তারের সাথে যোগাযোগ করতে হবে।

প্রাথমিক সেবার জন্য আপনি টেলিহেলথ্ নার্সের সাথেও যোগাযোগ করতে পারেন

যেভাবে এই পরিস্থিতি মোকাবিলা করতে হবে

- প্রতি ৪ ঘন্টা পর পর রক্তে গ্লুকোজের মাত্রা পরীক্ষা করতে হবে। লক্ষ্য হলো, রক্তে এই মাত্রা ৭-১০ মিলিমোল/লি এর মধ্যে রাখা।
- বার বার অল্প অল্প করে পানি পান করতে হবে।
- ডায়রিয়ার সময় চাল ধোয়া পানি খুব উপকারী।
- হাইপোগ্লাইসেমিয়া হলে মিষ্টি জুস এবং শুধু পানি বা ক্যালরিমুক্ত পানীয় পান করতে হবে।
- বমি এবং ডায়রিয়ার সময় মুখে খাওয়ার ওষুধ (যেমন: মেটফরমিন) সাময়িকভাবে বন্ধ রাখতে হবে।
- ইনসুলিন নেওয়া বন্ধ করা যাবে না তবে ডাক্তারের পরামর্শ মতো ডোজটি ঠিক করে নিতে হবে।
- অসুস্থতার দিনে শারীরিক ব্যায়াম বন্ধ রাখা উচিৎ।
- বেশি কিছু খেতে না পারলে, সম্ভব হলে সহজে হজম হয় এমন খাবার এবং পানীয় পান করতে হবে যাতে চিনি এবং ইলেক্ট্রোলাইট থাকে।

ওষুধের বিষয়ে যেকোনো সিদ্ধান্ত নেওয়ার জন্য ডাক্তারের সাথে যোগাযোগ করতে হবে।

রোগীকে হাসপাতালে নিয়ে যেতে হবে

- বমি বা ডায়রিয়া যদি ৬ ঘন্টার বেশি সময় ধরে চলতে থাকে।
- রক্তের গ্লুকোজের মাত্রা যদি ১৬.৭ মিলিমোল/লি এর বেশি হয়।
- হাইপোগ্লাইসেমিয়া হয়ে রোগী অজ্ঞান হয়ে গেলে।
- যদি শরীরের তাপমাত্রা ২৪ ঘন্টারও বেশি সময় ধরে ১০০° ডিগ্রি ফারেনহাইট থাকে।
- টানা ৩ দিন ধরে অসুস্থ থাকলে।
- পেটে ব্যথা, শ্বাসকষ্ট হলে।
- কিডনিজনিত সমস্যা বা হৃদরোগ থাকলে।

অনেক রাতে হঠাৎ সমস্যা হলে যদি ডাক্তারের সাথে যোগাযোগ করা সম্ভব না হয় তাহলে যেমন রাতে, অনুগ্রহ করে জরুরি বিভাগে যেতে হবে।

ডায়াবেটিসের জটিলতাসমূহ

ডায়াবেটিসের জটিলতাগুলো যেসব অঙ্গে দেখা দেয়

বড় রক্তনালী আক্রান্ত হওয়ার কারণে সৃষ্ট জটিলতা

জটিলতাগুলোতে জীবনের ঝুঁকি রয়েছে

স্ট্রোক

বছরে অন্তত একবার ক্যারোটিড ধমনী আল্ট্রাসাউন্ড পরীক্ষা করা প্রয়োজন ।
উচ্চ রক্তচাপের রোগীদের সতর্ক থাকতে হবে

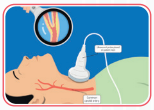

হার্ট অ্যাটাক

বিশেষ করে মেদবহুল ব্যক্তিদের (BMI ≥২৭.৫ কেজি/মি²) সচেতন হতে হবে। প্রয়োজনে বছরে একবার ইসিজি পরীক্ষা করতে হবে

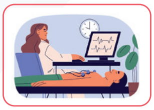

পেরিফেরাল ভাসকুলার ডিজিজ

জমাট বেঁধে থাকা রক্ত শরীরে সঠিকভাবে প্রবাহিত হতে পারে না। পরিস্থিতির অবনতি হলে অনেক সময় পা কেটে ফেলার প্রয়োজন হতে পারে।
নিয়মিত ব্যায়াম করতে হবে।

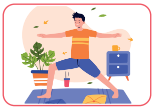

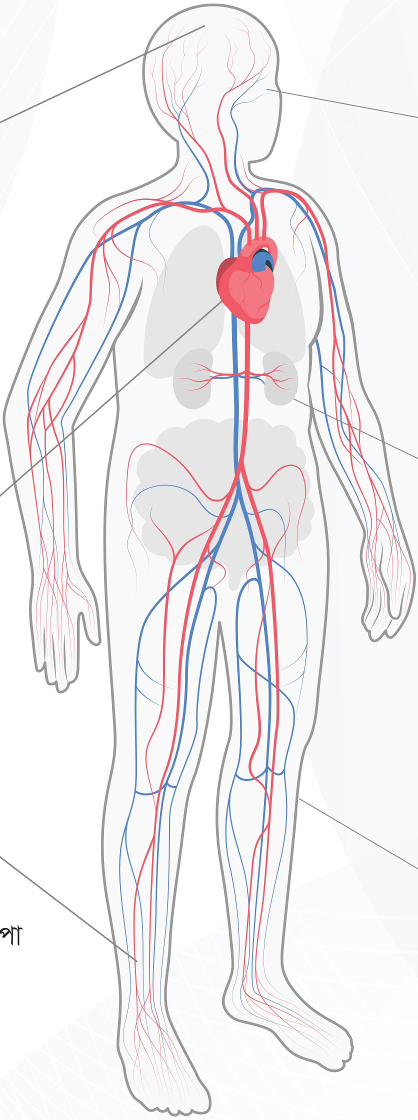

ছোট রক্তনালী আক্রান্ত হওয়ার কারণে সৃষ্ট জটিলতা

এই জটিলতাগুলো দৈনন্দিন জীবনে সমস্যা তৈরী করে

ডায়াবেটিক রেটিনোপ্যাথি

চোখে সমস্যা হয়
বছরে ১-২ বার চোখের ফান্ডাস পরীক্ষা করাতে হবে। নিয়মিত ডাক্তারের সাথে পরামর্শ করতে হবে।

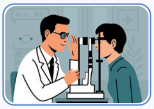

ডায়াবেটিক নেফ্রোপ্যাথি

কিডনিজনিত জটিলতা
এর কারণে ডায়ালাইসিস এবং হৃদযন্ত্রের ব্যর্থতা থেকে মৃত্যু হতে পারে। নিয়মিত রক্ত ও প্রস্রাব পরীক্ষা করাতে হবে কিডনির অবস্থা পরীক্ষা করার জন্য।

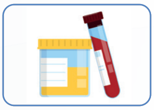

ডায়াবেটিক নিউরোপ্যাথি

পায়ের ক্ষত তৈরী হওয়া
প্রতিদিন অন্তত একবার পায়ের তালু, নখ ইত্যাদি পর্যবেক্ষণ।

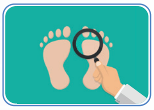

ডায়াবেটিক নেফ্রোপ্যাথির ধাপসমূহ ও চিকিৎসা:

ডায়াবেটিক নেফ্রোপ্যাথির প্রতিটি ধাপ সম্পর্কে জানা আবশ্যক এবং ধাপ অনুসারে ব্যবস্থা নিতে হবে।

কিডনির কার্যক্ষমতা পরীক্ষা এবং প্রস্রাব পরীক্ষার তথ্যের উপর ভিত্তি করে পর্যায়গুলি শ্রেণীবদ্ধ করা হয়।

পর্যায়	১	২	৩	৪	৫
কিডনির কার্যকারিতা= GFR (eGFR) (ml/min / 1.73 m²)	৩০ বা তার বেশি			৩০ এর কম	
প্রস্রাবে অ্যালবুমিনের মান (mg/gCr)	সাধারণ অ্যালবুমিনুরিয়া <৩০	মাইক্রোঅ্যালবুমিনুরিয়া ৩০-২৯৯	প্রকাশ্য অ্যালবুমিনুরিয়া ৩০০ বা তার বেশি	(প্রস্রাবের প্রোটিনের উপস্থিতি বা অনুপস্থিতি নির্বিশেষে)	
প্রস্রাবের প্রোটিনের মান (g/gCr)			অবিরাম প্রস্রাব প্রোটিন ০.৫ বা তার বেশি (১+)	→	
কিডনি ফাংশন বা কিডনির কার্যকারিতা	পূর্ব নেফ্রোপ্যাথি পর্যায় 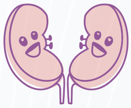	প্রাথমিক নেফ্রোপ্যাথি পর্যায় 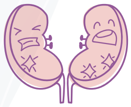	সুস্পষ্ট নেফ্রোপ্যাথি পর্যায় 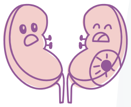	কিডনির অকার্যকারিতার পর্যায় 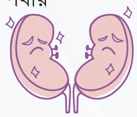	ডায়ালাইসিস চিকিৎসার পর্যায় 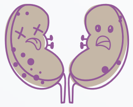
চিকিৎসা	ধূমপান নিষেধ	→	→	→	→
	লবণ ৫ গ্রাম/দিন	→	→	→	→
	"কার্বোহাইড্রেট বা শক্তি গ্রহণ: ২৫-৩০ ক্যালরি/কেজি/দিন (মেদবহূল হলেও: ২৫ ক্যালরি/কেজি/দিন নিতে পারবে)		→	→	→
			প্রোটিন: ০.৮-১.০ গ্রাম /কেজি/দিন	প্রোটিন: ০.৬-০.৮ গ্রাম/কেজি/দিন	
লক্ষ্য	BMI ২৫ এর নিচে	→	→	→	→
	ব্যায়াম থেরাপি	→	হার্টের কোন সমস্যা আছে কিনা তা দেখতে ডাক্তারের সাথে পরামর্শ নিতে হবে	→	→
	রক্তে গ্লুকোজের মাত্রা নিয়ন্ত্রণ; HbA1c ৭.০% এর নিচে		→	→	→
	রক্তচাপ/ব্লাড প্রেসার নিয়ন্ত্রণ:১৩০/৮০ মিলিমি মার্কারী এর নিচে		→	→	→
	লিপিড; LDL কোলেস্টেরল ১২০ মিলিগ্রাম/ডেল এর কম, এবং যদি হার্টের জটিলতা হয় সেক্ষেত্রে ১০০ মিলিগ্রাম/ডেল এর নিচে			→	→

সূত্র: জাপানিজ ডায়াবেটিস সোসাইটি ডায়াবেটিস চিকিৎসা নির্দেশিকা ২০১৬-২০১৭ এর উপর ভিত্তি করে তৈরি, বুনকোডো, ২০১৬ (পি৮২-৮৫) জাপান ডায়াবেটিস সোসাইটি ওয়েবসাইট, ডায়াবেটিক জয়েন্ট কমিটির রিপোর্ট (ডায়াবেটিক বৈশিষ্ট্যগুলির সংশোধন) দ্বারা প্রকাশিত।

[একজন ৭৭ বছর বয়সী ব্যক্তির জন্য চিকিৎসা প্রতিষ্ঠানে প্রাপ্ত পরীক্ষার রিপোর্টের উদাহরণ দেওয়া হলো]

Serum creatinine	1.0 mg/dl
eGFR	53.76 ml/min/1.73 m2
Serum Urea	28.38 mg/dl
Serum Uric Acid	4.80 mg/dl

eGFR; কিডনির পরিস্রাবণ ক্ষমতা পরীক্ষা করার জন্য একটি পরীক্ষা

[একজন ৫২ বছর বয়সী ব্যক্তির জন্য চিকিৎসা প্রতিষ্ঠানে প্রাপ্ত পরীক্ষার রিপোর্টের উদাহরণ দেওয়া হলো]

Serum creatinine	2.64 mg/dl
eGFR	18.99 ml/min/1.73 m2
Serum Urea	59.39 mg/dl
Serum Uric Acid	8.10 mg/dl

কিডনির কার্যকারিতা ১০% হলে প্রায়ই ডায়ালাইসিস প্রয়োজন হয়।

দ্বিতীয় রিপোর্টে দেখা যাচ্ছে সিরাম ক্রিয়েটিনিন (Serum creatinine) -এর মান বেশি হওয়ার কারণে কিডনির কার্যকারিতা (eGFR) অনেক কমে গিয়েছে ।

নিম্নলিখিত পরীক্ষার মানগুলি একটি কর্মক্ষম কিডনির স্বাভাবিক মানদন্ড।

পরীক্ষার নাম	স্বাভাবিক মানদন্ড
Serum Creatinine	0.55-1.3 mg/dl
BUN	0.7-21.0 mg/dl
Potassium	3.5-5.2 mEq/l
Sodium	136-148 mEq/l
Chloride	98-108 mEq/l
Calcium	8.5-10.03 mEq/l
eGFR	>60 ml/min/1.73 m2

সূত্র: বি.আই.এইচ.এস জেনারেল হাসপাতাল ল্যাব রেকর্ড।

অ্যান্টিডায়াবেটিক ঔষধসমূহ

Biguanide drugs:
(Metformin)

লিভার বা যকৃতে নতুনভাবে গ্লুকোজ উৎপাদনকে কমায়। একই সময়ে মাংসপেশীতে গ্লুকোজ ব্যবহারকে বাড়ায়।

Rapid-acting insulin secretion promoter
(glinide drug)

খাওয়ার পরপরই দ্রুত ইনসুলিন নিঃসরণে সাহায্য করে। এর প্রভাব ক্ষণস্থায়ী। পোষ্টপ্রান্ডিয়াল হাইপারগ্লাইসেমিয়ার ক্ষেত্রে এটি ভালো কাজ করে।

Thiazolidinediones
(Pioglitazone, rosiglitazone)

রক্তে গ্লুকোজের মাত্রা নিয়ন্ত্রণে কঙ্কালপেশী এবং লিভারে ইনসুলিনের কার্যক্ষমতা বাড়ায়।

Alpha-glucosidase inhibitor (∞-G1):
(Acarbose, miglitol, voglibose)

খাদ্য পরিপাকে সাহায্যকারী এনজাইমের কার্যক্ষমতা কমিয়ে গ্লুকোজের শোষণে দেরি করায়। এজন্য এই ঔষধটি ঠিক খাবার খাওয়ার আগে খেতে হয় যেন ঔষধ এবং খাবার ভালোভাবে মিশে সঠিক প্রভাব ঘটাতে পারে।

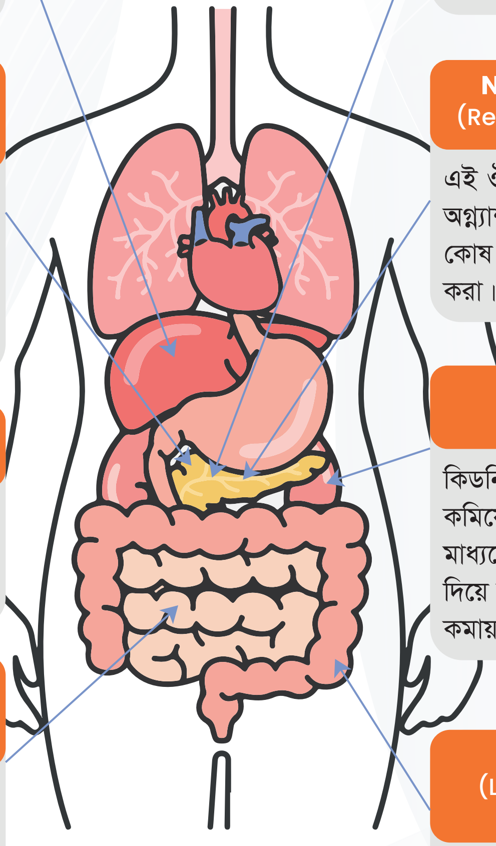

Sulfonylurea (SU) drugs
(Glipizide, gliclazide, glimepiride, glibenclamide)

এগুলো বিটা কোষ থেকে ইনসুলিন নিঃসরণ করার জন্য সরাসরি অগ্ন্যাশয়কে উদ্দিপীত করে।

Non-sulfonylureas:
(Repaglinide, Nateglinide)

এই ঔষধগুলোরও কাজ হলো অগ্ন্যাশয়কে উদ্দিপীত করে বিটা কোষ থেকে ইনসুলিন নিঃসরণ করা।

SGLT2 inhibitor:
(Empagliflozin, etc.)

কিডনিতে গ্লুকোজের পুনঃশোষণ কমিয়ে সেই গ্লুকোজকে প্রস্রাবের মাধ্যমে দেহের বাহিরে বের করে দিয়ে রক্তে গ্লুকোজের মাত্রা কমায়।

DPP-4 inhibitor:
(Linagliptin, alogliptin, sitagliptin, etc.)

রক্তে গ্লুকোজের মাত্রা অনুযায়ী ইনসুলিন নিঃসরণে সাহায্য করে। সেই সাথে গ্লুকাগন নামক হরমোনের নিঃসরণ কমায় যা রক্তে গ্লুকোজের মাত্রা বাড়ায়।

সূত্র: ডায়াবেটিস মেলাইটাসের জাতীয় গাইডলাইন/নির্দেশিকা; প্রথম সংস্করণ, আগস্ট, ২০২৩।

দ্রুত কার্যকরী ইনসুলিন

Insulin Lispro (ইনসুলিন লিসপ্রো)
Insulin Aspart (ইনসুলিন এসপার্ট)
Insulin Glulisine (ইনসুলিন গ্লুলাইসিন)

দীর্ঘমেয়াদী ইনসুলিন

Insulin Detemir
(ইনসুলিন ডেটেমির)
Insulin Glarine
(ইনসুলিন গ্লেরিন)
Insulin Degludec
(ইনসুলিন ডেগ্লুডেক)

মিশ্র ইনসুলিন (৩০/৭০, ৫০/৫০, ২৫/৭৫)

Biphasic Human Insulin
(বাইফেজিক হিউম্যান ইনসুলিন),
Biphasic Insulin Aspart
(বাইফেজিক ইনসুলিন এসপার্ট)
Biphasic Insulin Lispro
(বাইফেজিক ইনসুলিন লিসপ্রো)

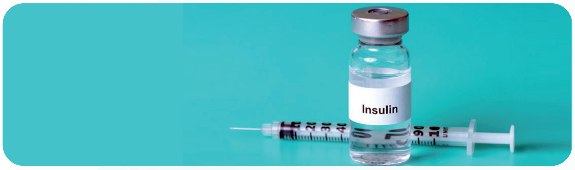

সূত্র: ডায়াবেটিস মেলাইটাসের জাতীয় গাইডলাইন/নির্দেশিকা; প্রথম সংস্করণ, আগস্ট, ২০২৩।

ঔষধ খাওয়ার তিনটি নিয়ম

প্রথমে, ঔষধ এবং তার সঠিক ডোজ এবং সময় সম্পর্কে জানতে হবে।

১. ঔষধের সঠিক ডোজ এবং সময় মনে রাখতে হবে

ঔষধ খেতে ভুলে যাওয়া যাবে না।

তবে যদি এমন হয় যে প্রায়ই সময়মত ঔষধ খেতে ভুলে যাওয়ার ঘটনা ঘটছে, তাহলে ডাক্তারের সাথে পরামর্শ করে নিজ জীবনধারা অনুসারে ডোজ পরিবর্তন করে নিতে হবে।

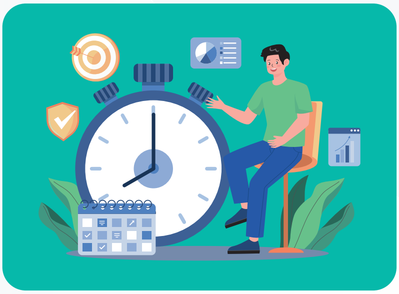

নিয়মিত ঔষধ খাওয়ার কথা মনে রাখার কয়েকটি উপায়-

- ঔষধগুলি একটি পাত্রে দৃশ্যমান জায়গায় রাখতে হবে।
- ফোনে একটি অ্যালার্ম সেট করে রাখা ভালো।
- নোটবুক এবং ক্যালেন্ডারে দিন, তারিখ এবং সময় চিহ্নিত করে রাখা।
- পরিবারের একজনকে নিয়মিত ঔষধ খাওয়ার কথা মনে করিয়ে দিতে বলা যেতে পারে।

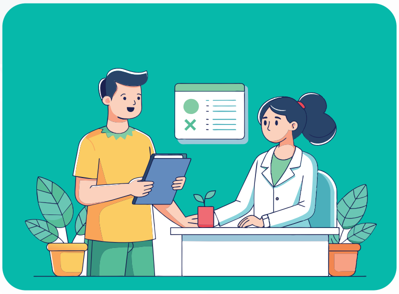

২. ডাক্তারের নির্দেশ অনুসারে ঔষধ খেতে হবে।

৩. চিকিৎসার মধ্যে কোনো বিরতি না দিয়ে নিয়মিত ডাক্তারের ভিজিট করতে হবে।
