# Supplementary material for: Telenursing Health Education and Lifestyle Modification Among Patients With Diabetes in Bangladesh: Protocol for a Pilot Study With a Quasi-experimental Pre- and Postintervention Design
Source: JMIR Res Protoc. 2025 May 9;14:e71849. doi: 10.2196/71849 (PMC12102625; doi:10.2196/71849)
Supplement: Multimedia Appendix 3 [file resprot_v14i1e71849_app3.pdf]

Diabetes Mellitus

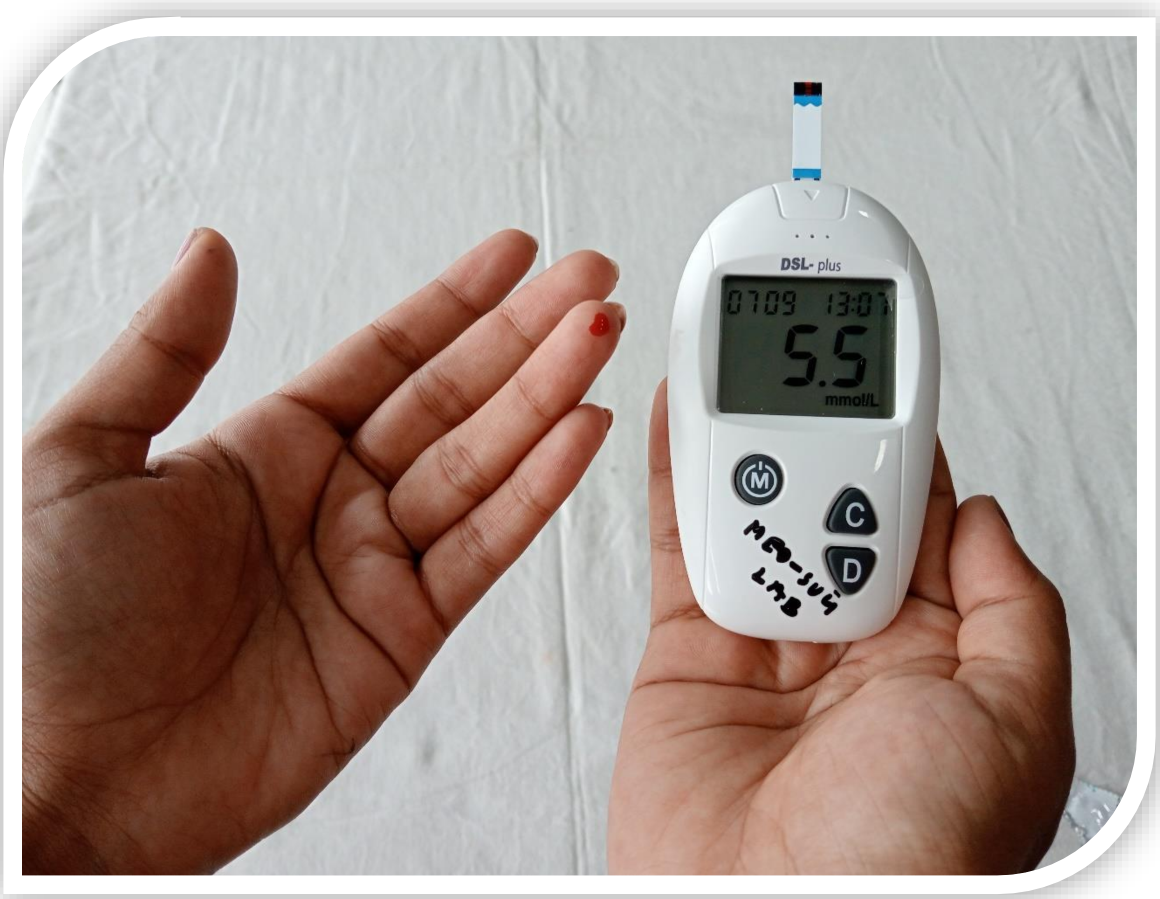

You are not alone

We can help to slow the progression of your disease

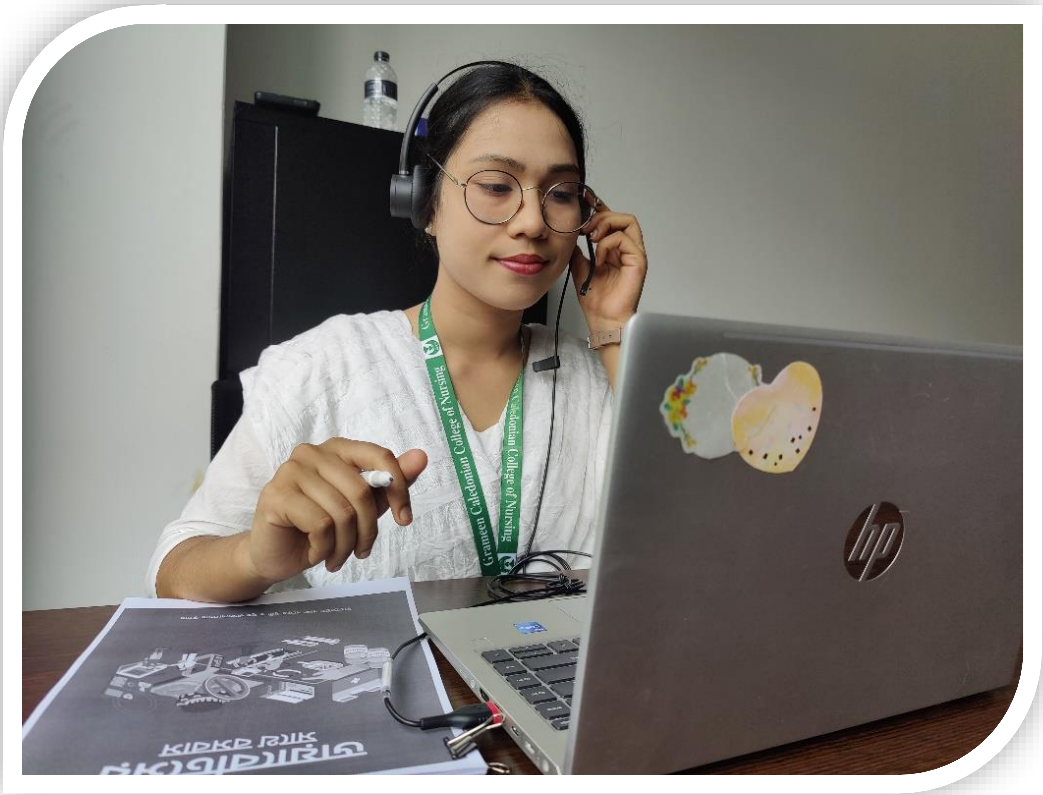

Name:

Telenurse Service Officer

My Information

Name	
Age	
Gender	
Marital status	
Mobile No.	
Address	

Who will Support me in my daily recovery?

I want to be good for this person. Write down what you want to tell that person and what you want them to do together to help you get better.

....

....

....

....

....

....

Life goals

Why do you want to get better?

(In order for people with illness to be treated positively, it is important to have clear goals in life)

....

....

....

....

....

....

Diseases you have...

☐ Obesity (please put your BMI here………)

☐ Hypertension

☐ Dyslipidemia (Hypercholesterolemia)

☐ Diabetes

☐ Diabetic retinopathy

☐ Diabetic neuropathy

☐ Kidney disease

☐ Myocardial infarction and angina pectoris

☐ Stroke

☐ Arrythmia

☐ Fatty liver/ alcoholic liver disease

☐ Anemia

☐ Hyperkalemia

☐ Hyperuricemia

☐ 0thers (……………………………………………………………………………………)

What I am working on

- Meals (3 meals regularly and don't eat too much) Yes No
- Salt (gm/day)
- Energy intake (Kcal/day)
- Protein intake (gm/day)
- Water intake (ml/day)
- Oral medication/Injection Yes No
- Cessation of Smoking Yes No
- Exercise ( )
- Foot care (Daily/weekly)
- Self-monitoring blood sugar (morning /noon / evening)
- Blood pressure measurement (twice in a week …..….mmHg)
- Weight measurement (twice a week, current weight………………kg)
- Preventing complication
- Don`t get stressed out
- Ophthalmology (examination once a month)
- Dental visit
- Others ( )

Treatment and test schedule and test results

Inspection Item	Standard Value	Unit	Date	Date	Date	Remarks
HbA1c	<6.5%					
FBS (fasting blood sugar)	6.1-7.0	mmol/l				
RBG	7.8-10.0	mmol/l				
Total cholesterol	Up to 200	mg/dl				
LDL cholesterol	<150	mg/dl				
HDL cholesterol	M:>40, F:>50	mg/dl				
Triglyceride	50-150	mg/dl				
Serum Creatinine	0.55-1.3	mg/dl				
e-GFR	>60	ml/min/1.73 m2				
Urine Protein	0-14	mg/dl				
Hemoglobin	M:13-18<br>F: 11.5-16.5	g/dl				
Blood Group	……	………..				

Observe the sole of your feet:

If you notice any abnormalities such as small scratches, calluses, corns, athlete's foot, sores or numbness consult your doctor

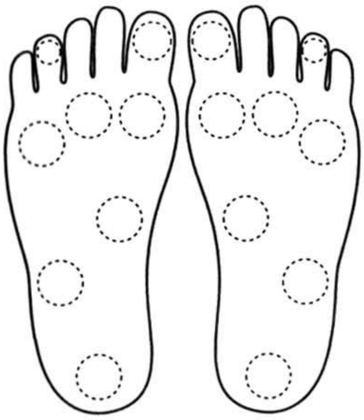

1 month

Month:................

Date:................

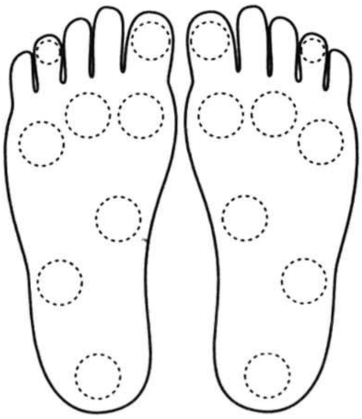

2 month

Month:.............

Date:................

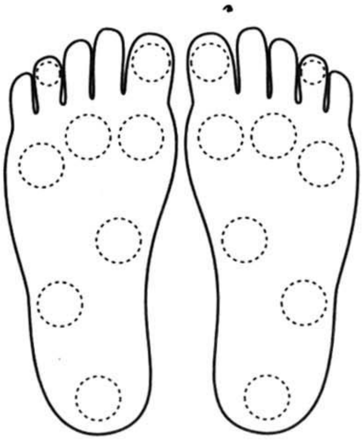

3 month

Month:............

Date:................

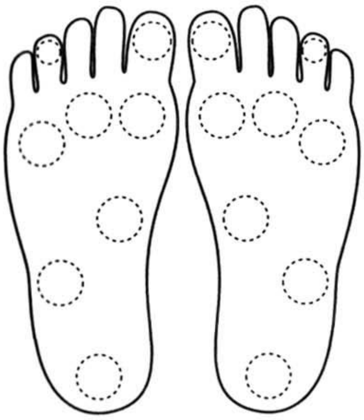

4 month

Month:.............

Date:................

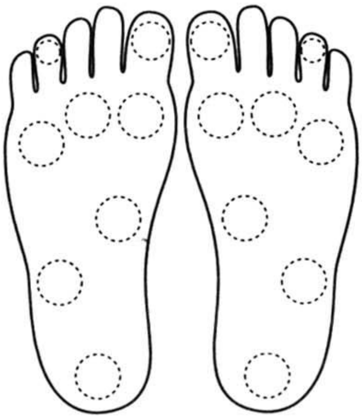

5 month

Month:.............

Date:................

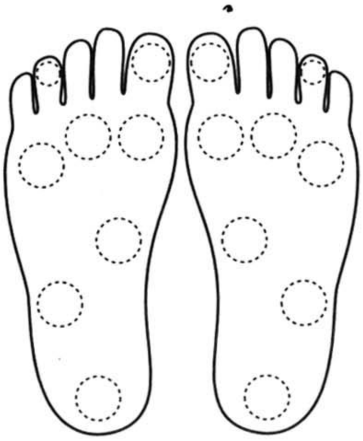

6 month

Month:.............

Date:................

Daily Note: Month:

Monthly goal:

Day count	RBS	Blood Pressure	Goal		medication	Others
			Meal	Exercise		
1st						
2nd						
3rd						
4th						
5th						
6th						
7th						
8th						
9th						
10th						
11th						
12th						
13th						
14th						
15th						

Day count	RBS	Blood Pressure	Goal		medication	Others
			meal	exercise		
16th						
17th						
18th						
19th						
20th						
21st						
22nd						
23rd						
24th						
25th						
26th						
27th						
28th						
29th						
30th						
31st						

Daily Note: Month:

Monthly goal:

Day count	RBS	Blood Pressure	Goal		medication	Others
			Meal	Exercise		
1st						
2nd						
3rd						
4th						
5th						
6th						
7th						
8th						
9th						
10th						
11th						
12th						
13th						
14th						
15th						

Day count	RBS	Blood Pressure	Goal		medication	Others
			meal	exercise		
16th						
17th						
18th						
19th						
20th						
21st						
22nd						
23rd						
24th						
25th						
26th						
27th						
28th						
29th						
30th						
31st						

Daily Note: Month:

Monthly goal:

Day count	RBS	Blood Pressure	Goal		medication	Others
			Meal	Exercise		
1st						
2nd						
3rd						
4th						
5th						
6th						
7th						
8th						
9th						
10th						
11th						
12th						
13th						
14th						
15th						

Day count	RBS	Blood Pressure	Goal		medication	Others
			meal	exercise		
16th						
17th						
18th						
19th						
20th						
21st						
22nd						
23rd						
24th						
25th						
26th						
27th						
28th						
29th						
30th						
31st						

Daily Note: Month:

Monthly goal:

Day count	RBS	Blood Pressure	Goal		medication	Others
			Meal	Exercise		
1st						
2nd						
3rd						
4th						
5th						
6th						
7th						
8th						
9th						
10th						
11th						
12th						
13th						
14th						
15th						

Day count	RBS	Blood Pressure	Goal		medication	Others
			meal	exercise		
16th						
17th						
18th						
19th						
20th						
21st						
22nd						
23rd						
24th						
25th						
26th						
27th						
28th						
29th						
30th						
31st						

Daily Note: Month:

Monthly goal:

Day count	RBS	Blood Pressure	Goal		medication	Others
			Meal	Exercise		
1st						
2nd						
3rd						
4th						
5th						
6th						
7th						
8th						
9th						
10th						
11th						
12th						
13th						
14th						
15th						

Day count	RBS	Blood Pressure	Goal		medication	Others
			meal	exercise		
16th						
17th						
18th						
19th						
20th						
21st						
22nd						
23rd						
24th						
25th						
26th						
27th						
28th						
29th						
30th						
31st						

Daily Note: Month:

Monthly goal:

Day count	RBS	Blood Pressure	Goal		medication	Others
			Meal	Exercise		
1st						
2nd						
3rd						
4th						
5th						
6th						
7th						
8th						
9th						
10th						
11th						
12th						
13th						
14th						
15th						

Day count	RBS	Blood Pressure	Goal		medication	Others
			meal	exercise		
16th						
17th						
18th						
19th						
20th						
21st						
22nd						
23rd						
24th						
25th						
26th						
27th						
28th						
29th						
30th						
31st						

Daily Note: Month:

Monthly goal:

Day count	RBS	Blood Pressure	Goal		medication	Others
			Meal	Exercise		
1st						
2nd						
3rd						
4th						
5th						
6th						
7th						
8th						
9th						
10th						
11th						
12th						
13th						
14th						
15th						

Day count	RBS	Blood Pressure	Goal		medication	Others
			meal	exercise		
16th						
17th						
18th						
19th						
20th						
21st						
22nd						
23rd						
24th						
25th						
26th						
27th						
28th						
29th						
30th						
31st						

Check change in your blood sugar level

What kind of meal raise your blood sugar levels and when is the blood sugar level highest after meal?

……………………………………………………………………….

Date	Time eaten	What I eat	Fasting	Immediately After eating	1 hour later	2 hours later
	Morning<br><br>evening					
	Morning<br><br>evening					
	Morning<br><br>evening					
	Morning<br><br>evening					
	Morning<br><br>evening					
	Morning<br><br>evening					
	Morning<br><br>evening					
	Morning<br><br>evening					
	Morning<br><br>evening					
	Morning<br><br>evening					
	Morning<br><br>evening					
	Morning<br><br>evening					
	Morning<br><br>evening					
	Morning<br><br>evening					
	Morning<br><br>evening					
	Morning<br><br>evening					

	Morning<br><br>evening					
	Morning<br><br>evening					
	Morning<br><br>evening					
	Morning<br><br>evening					
	Morning<br><br>evening					
	Morning<br><br>evening					
	Morning<br><br>evening					
	Morning<br><br>evening					
	Morning<br><br>evening					
	Morning<br><br>evening					
	Morning<br><br>evening					
	Morning<br><br>evening					

Let's review the daily schedule

(My day)

Current

Time	Activity	Time	Activity
5.00 am		3.00 pm	
6.00 am		4.00 pm	
7.00 am		5.00 pm	
8.00 am		6.00 pm	
9.00 am		7.00 pm	
10.00 am		8.00 pm	
11.00 am		9.00 pm	
12.00 pm		10.00 pm	
1.00 pm		11.00 pm	
2.00 pm		12.00 am	

Let’s make a change

Daily schedule (target)

Time	Activity	Time	Activity
5.00 am		3.00 pm	
6.00 am		4.00 pm	
7.00 am		5.00 pm	
8.00 am		6.00 pm	
9.00 am		7.00 pm	
10.00 am		8.00 pm	
11.00 am		9.00 pm	
12.00 pm		10.00 pm	
1.00 pm		11.00 pm	
2.00 pm		12.00 am	

If you can change your mind

You can change your life

Telenursing
