# Supplementary material for: Telenursing Health Education and Lifestyle Modification Among Patients With Diabetes in Bangladesh: Protocol for a Pilot Study With a Quasi-experimental Pre- and Postintervention Design
Source: JMIR Res Protoc. 2025 May 9;14:e71849. doi: 10.2196/71849 (PMC12102625; doi:10.2196/71849)
Supplement: Multimedia Appendix 4 [file resprot_v14i1e71849_app4.docx]

| **Name of the Client:** | **Gender:** | **Age:** | | **Height:** |
| --- | --- | --- | --- | --- |
| **Id No:** |  | | | |
| **Data collected** | **Initial** | **Month:** | | |
| **Week** |  | **2nd Week** | **3rd Week** | **4th Week** |
| **Date** |  |  |  |  |
| Compliance with regular hospital visits |  |  |  |  |
| Name of the institution visited: |  |  |  |  |
| Reason for the hospital visit |  |  |  |  |
| Number of non-regular hospital visits |  |  |  |  |
| Reason for non-regular hospital visits |  |  |  |  |
| Number of hospitalization days |  |  |  |  |
| Number of missed work due to illness |  |  |  |  |
| **Subjective Symptoms/test report data** |  |  |  |  |
| Weight (kg) |  |  |  |  |
| BMI |  |  |  |  |
| Blood pressure (mmHg) |  |  |  |  |
| RBS/FBS/2HABS |  |  |  |  |
| **Lab data** |  |  |  |  |
| Total Cholesterol: T-C (mg/dl) |  |  |  |  |
| HDL-C (mg/dl) |  |  |  |  |
| LDL-C (mg/dl) |  |  |  |  |
| Natural Fat: TG (mg/dl) |  |  |  |  |
| S. Creatinine |  |  |  |  |
| Urine Albumin |  |  |  |  |
| HBA1c (%) |  |  |  |  |
| **Percentage of implementation of action goals (enter in % Percentage of days in a month when the goals were implemented)** | | | | |
| Diet therapy implementation rate (day/M) |  |  |  |  |
| Exercise therapy implementation rate (day/M) |  |  |  |  |
| Drug therapy implementation rate (day/M) |  |  |  |  |
| **Changes in treatment policy, drug, etc.** |  |  |  |  |
| Diet remedy: Calorie content |  |  |  |  |
| Salt content |  |  |  |  |
| Protein quantity |  |  |  |  |
| Other menus |  |  |  |  |
| Exercise therapy |  |  |  |  |
| Drug therapy |  |  |  |  |
| **Understanding treatment and confidence level** |  |  |  |  |
| Understanding the content of health education book |  |  |  |  |
| Understanding the self-monitoring book |  |  |  |  |
| Sense of control over one's life |  |  |  |  |
| Controlling emotion regarding illness |  |  |  |  |
| Intensity of social activity |  |  |  |  |
| **Report from the nurse to the attending physician** |  |  |  |  |
