# Supplementary material for: Telenursing Health Education and Lifestyle Modification Among Patients With Diabetes in Bangladesh: Protocol for a Pilot Study With a Quasi-experimental Pre- and Postintervention Design
Source: JMIR Res Protoc. 2025 May 9;14:e71849. doi: 10.2196/71849 (PMC12102625; doi:10.2196/71849)
Supplement: Multimedia Appendix 5 [file resprot_v14i1e71849_app5.docx]

Multimedia Appendix 5

The outcome table and data collection schedule.

| Items | | Baseline (month 0) | Month 1 | Month 2 | Month 3 (midline) | Month 4 | Month 5 | Month 6 (endline) |
| --- | --- | --- | --- | --- | --- | --- | --- | --- |
| Sociodemographic | | ✓ |  |  |  |  |  |  |
| Clinical history | | ✓ |  |  |  |  |  |  |
| Lifestyle | | ✓ |  |  |  |  |  |  |
| **Primary outcome** | | | | | | | | |
|  | Change in HbA_1c_^a^ | ✓ |  |  | ✓ |  |  | ✓ |
| **Secondary outcomes** | | | | | | | | |
| **Operational feasibility of the system (qualitative)** | | | | | | | | |
|  | Interview with the nurses, PHC^b^ staff |  |  |  |  |  |  | ✓ |
|  | Any issues and obstacles | ✓ | ✓ | ✓ | ✓ | ✓ | ✓ | ✓ |
| **Patient feasibility (qualitative and quantitative)** | | | | | | | | |
|  | Engagement rate | ✓ |  |  |  |  |  |  |
|  | Follow-up rate |  | ✓ | ✓ | ✓ | ✓ | ✓ | ✓ |
|  | Satisfaction | ✓ | ✓ | ✓ | ✓ | ✓ | ✓ | ✓ |
|  | Nurse education (any revision) | ✓ | ✓ | ✓ | ✓ | ✓ | ✓ | ✓ |
|  | Education booklet used (any revision) | ✓ | ✓ | ✓ | ✓ | ✓ | ✓ | ✓ |
|  | Self-monitoring booklet used (any revision) | ✓ | ✓ | ✓ | ✓ | ✓ | ✓ | ✓ |
|  | Material used (any revision) | ✓ | ✓ | ✓ | ✓ | ✓ | ✓ | ✓ |
| **Behavior modification (quantitative and qualitative)** | | | | | | | | |
|  | Achievement of monthly set goals | ✓ | ✓ | ✓ | ✓ | ✓ | ✓ | ✓ |
|  | Behavior change (scale) | ✓ | ✓ | ✓ | ✓ | ✓ | ✓ | ✓ |
| **Self-efficacy** | | |  |  | ✓ | ✓ | ✓ | ✓ |
|  | Self-efficacy scale | ✓ | ✓ | ✓ | ✓ | ✓ | ✓ | ✓ |
| **Complication management** | | |  |  | ✓ | ✓ | ✓ | ✓ |
|  | Hospitalized by complication | ✓ | ✓ | ✓ | ✓ | ✓ | ✓ | ✓ |
|  | Newly diagnosed (any) | ✓ | ✓ | ✓ | ✓ | ✓ | ✓ | ✓ |
|  | Medication and treatment change | ✓ | ✓ | ✓ | ✓ | ✓ | ✓ | ✓ |
|  | Death, any adverse events | ✓ | ✓ | ✓ | ✓ | ✓ | ✓ | ✓ |
|  | Eye check (clinic visit) (Retinopathy development) | ✓ | ✓ | ✓ | (ask within an initial 3 months, a patient visits and check them) | | | |
|  | ECG (Cardiovascular check) | ✓ | ✓ | ✓ |  |  |  |  |
|  | Laboratory test (Cholesterol, renal) | ✓ | ✓ | ✓ |  |  |  |  |
| **Physical and laboratory data** | | |  |  |  | | | |
|  | Rate of patients who have achieved HbA_1c_ in the recommended level |  |  |  |  |  |  | ✓ |
|  | FBS^c^ or RBS^d^ | ✓ | ✓ | ✓ | ✓ | ✓ | ✓ | ✓ |
|  | Blood pressure value (SBP^e^ and DBP^f^) | ✓ | ✓ | ✓ | ✓ | ✓ | ✓ | ✓ |
|  | BMI | ✓ | ✓ | ✓ | ✓ | ✓ | ✓ | ✓ |
|  | Non-HDL^g^ cholesterol (TC^h^, HDL-c^i^), Triglyceride | ✓ | ✓ | ✓ | ✓ | ✓ | ✓ | ✓ |
|  | eGFR^j^ (Creatinine) BUN^k^ for Stage 4 | ✓ |  |  | ✓ (Stage 4) |  |  | ✓ |
| **Economical evaluation** | | |  |  |  | | | |
|  | Cost-effectiveness |  |  |  |  |  |  | ✓ |

^a^HbA_1c_: Hemoglobin A1c

^b^PHC: Primary Health Center

^c^FBS: fasting blood sugar

^d^RBS: random blood sugar

^e^SBP: systolic blood pressure

^f^DBP: diastolic blood pressure

^g^HDL: high-density lipoprotein

^h^TC: total cholesterol

^i^HDL-c: high-density lipoprotein cholesterol

^j^eGFR: estimated glomerular filtration rate

^k^BUN: Blood Urea Nitrogen
